# Vibrational Spectroscopic and Quantum-Chemical Study of Indole–Ketone Hydrogen-Bonded Complexes

**DOI:** 10.3390/molecules30132685

**Published:** 2025-06-21

**Authors:** Branislav Jović, Nataša Negru, Dušan Dimić, Branko Kordić

**Affiliations:** 1 Faculty of Sciences, University of Novi Sad, Trg Dositeja Obradovića 3, 21000 Novi Sad, Serbia; 2 Faculty of Physical Chemistry, University of Belgrade, Studentski Trg 12-16, 11000 Belgrade, Serbia

**Keywords:** indole, hydrogen bond, ketones, FTIR, DFT, QTAIM

## Abstract

This study investigates the structural and energetic properties of hydrogen-bonded complexes between indole and a range of aliphatic, cyclic, and aromatic ketones using a combined vibrational spectroscopic and quantum-chemical approach. FTIR measurements in CCl_4_ revealed redshifts in the N-H stretching vibration of indole upon complexation, with formation constants (K_a_) ranging from 0.3 to 6.6 M^−1^. Cyclohexanone displayed the strongest binding, while benzophenone exhibited the weakest interaction. Quantum-chemical calculations, employing CREST and MMFF94 conformational sampling, along with M06-2X/6-311++G(d,p) optimizations, confirmed the formation of hydrogen bonds and additional weak interactions that govern the stability of the complex. QTAIM analysis revealed moderate closed-shell hydrogen bonds with electron densities at the bond critical points (ρ) ranging from 0.010 to 0.019 a.u. and potential energy densities (V) from −18.4 to −36.4 kJ mol^−1^. Multivariate regression analysis established strong correlations (R^2^ = 0.928 and 0.957) between experimental binding constants and theoretical descriptors, including binding energy, NBO charge on oxygen atom, ionization potential, and electrophilicity index, highlighting the interplay between geometric, electronic, and global reactivity factors. This comprehensive study underlines the predictive power of spectroscopic and quantum descriptors for assessing hydrogen bonding in biologically relevant systems.

## 1. Introduction

The role of indole and indole-type molecules in biochemical processes remains a significant area of study. Examining the amino acid residues of proteins and the mechanisms of enzymatic reactions requires further clarification and expanded knowledge regarding indole. From a fundamental spectrochemical perspective, indole is particularly interesting due to its planar π-electronic system and N-H proton donor group, which allow for various interactions. Additionally, the carbonyl group represents an indispensable structural segment of many biochemical macromolecules, and the potential interactions with this group continue to be an important field of research in terms of fundamentals and applications.

Since the indole ring represents a side chain of the amino acid tryptophan, it is an integral part of proteins. Transmembrane proteins and peptides are known to have an exceptionally high content of tryptophan. Its ability to form hydrogen bonds significantly influences protein structures within cell membranes, where the indole ring interacts with other molecules, especially the abundant (lipid) carbonyl group. This amino acid participates in both hydrophobic and hydrophilic interactions, which explains its role in the transmembrane space and its capacity to form hydrogen bonds [1]. Furthermore, indole ring-containing compounds exhibit a wide range of biological activities, such as antidepressant, antifungal, antimicrobial, antiviral, anticancer, antihypertensive, anti-HIV, and more [2]. Therefore, it is a favorable structural motif in many medical therapeutics [3,4,5,6,7,8,9]. Aside from pharmaceuticals, indole and its derivatives have been used in solar cell production and similar applications [10,11,12,13,14,15,16]. The indole ring is a suitable electron donor because of its aromatic structure and the presence of nitrogen atoms [17]. Recent research reported novel use of its charge transfer properties in organic synthesis [18,19,20]. Studying the intermolecular interactions of indoles is also very important for understanding similar bioactive molecules, such as pyrrole [21,22,23].

The significance of the N-H···O hydrogen bond interaction in the crystal structure of indole derivatives has been documented [24]. While various crystal symmetries have been investigated, the hydrogen bond formed between the indole N-H group and a carbonyl group has emerged as a dominant intermolecular interaction that significantly influences the crystal packing. Further, N-H···O hydrogen bonding between polymer chains of the poly (iminoimino ketone ketone) has been reported [25]. The good thermal stability of poly (iminoimino ketone ketone) compared to similar polymers can be attributed to the formation of this hydrogen bond. Therefore, determining the physicochemical characteristics of N-H···O intermolecular interactions could provide better insights into the structure and potential modifications of polymer materials of this type.

**Figure 1:**
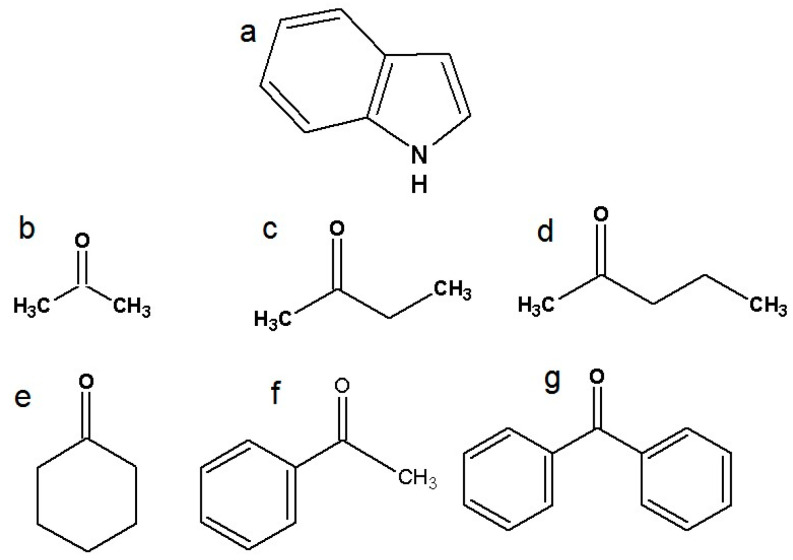
Structural formulas of investigated molecules: (**a**) indole, (**b**) acetone, (**c**) 2-butanone, (**d**) 2-pentanone, (**e**) cyclohexanone, (**f**) acetophenone, and (**g**) benzophenone.

There have been many experimental and computational studies concerning indole intermolecular interactions via the N-H group, but not with specific carbonyl compounds [26,27,28,29]. Further investigation of indole hydrogen bonding and charge transfer complexes is needed. This study represents a continuation of our ongoing systematic research of indoles and amides as important model systems for biological molecules [30,31,32]. The structures of indole and six ketones are presented in Figure 1. The ketones were chosen to investigate the effects of the alkyl chain, aliphatic ring, and aromatic systems, while also considering the influence of steric factors. Other polar groups in the proton acceptor molecules besides the carbonyl group were avoided to ensure that only one type of hydrogen bond can form between the proton donor and the acceptor, in accordance with the limitations of Becker’s method [33].

## 2. Results and Discussion

### 2.1. FTIR Spectroscopic Analysis

**Figure 2:**
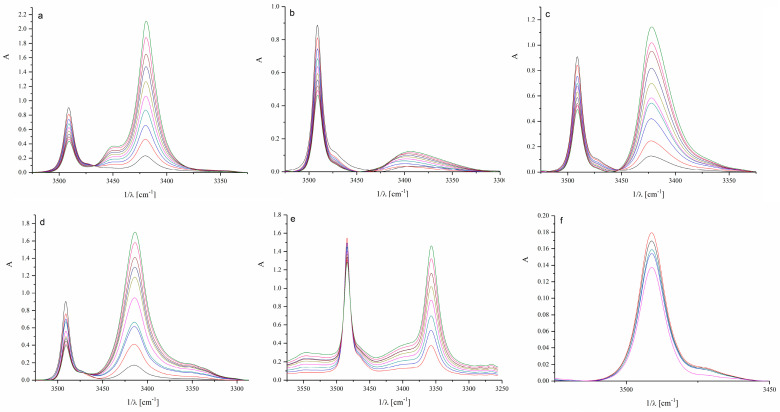
Infrared spectra of investigated systems: (**a**) acetone, (**b**) 2-butanone, (**c**) 2-pentanone, (**d**) cyclohexanone, (**e**) acetophenone, and (**f**) benzophenone. (The different colors of the spectra represent increasing concentrations of ketones in the presence of indole in the range of 0.01 to 0.3 M).

Spectroscopic parameters for N-H···O hydrogen-bonded complexes and the equilibrium constants from infrared spectroscopy (IR) experiments are summarized in Table 1, where Δν(NH) and ν_1/2_ are wavenumber shift and halfwidth of the N-H complex band, respectively. Figure 2 relates to the IR spectra of indole in the presence of different ketones. The wavenumber shift represents the difference between the N-H group band position of the free indole and the band position of the same indole N-H group hydrogen-bonded to a ketone.

As shown in the data from Figure 2 and Table 1, lower alkyl ketones form similar hydrogen-bonded complexes with indole regarding structure and stability, whereas cyclic ketones and aromatics behave somewhat differently. The band of the free, non-bonded N-H indole group appears at 3491 cm^−1^ (Figure S1), while the band of the hydrogen-bonded N-H group appears at lower wavenumbers. The most noticeable difference occurs in complexes with the aromatic benzophenone. In the case of benzophenone, two bulky phenyl rings limit the accessibility of the carbonyl group for direct hydrogen bonding with the N-H group of indole and favor other types of interactions. It can be assumed that π–π interactions prevail in the case of acetophenone and benzophenone. Cyclohexanone exhibits a higher stability constant than the other tested ketones, which could be attributed to the sterically accessible carbonyl group of cyclohexanone (Table 1).

If the strengths of the obtained complexes of indole with ether and carbonyl oxygen are compared, the following can be observed: indole with different ethers, both cyclic and acyclic, forms complexes with hydrogen bond constants ranging from 1.5 to 3 M^−1^, which are significantly lower than the constants in complexes with ketones. The greater stability of the indole complex with ketones is likely a consequence of the higher polarity of the carbonyl oxygen compared to the ether group. Hydrogen bonding of indole with aromatic π-systems has been reported. Muñoz et al. [34] obtained values for formation constants for hydrogen-bonded complexes of indole with different aromatic molecules in the range of 0.3–0.9 dm^3^ mol^−1^. As may be expected, these values are much lower than those obtained for systems with ketones in this work.

The interaction of the carbonyl group of ketones has been a research subject for a long time [35] and remains relevant. Specifically, in the spectroscopic examination of the complexes formed by acetone, acetophenone, and benzophenone with phenol derivatives, similar to this study, it was determined that, due to steric hindrances, the most stable complexes with phenols are formed with acetone, followed by acetophenone and benzophenone. More recent studies [36] on the interactions of the carbonyl group using spectroscopic methods include exploring the interactions of ketone carbonyl groups with alcohols. Interestingly, and similar to the results obtained in this study, it has been observed that the redshifts of the carbonyl band of ketones are greater for cyclic ketones than for acyclic ones. It was found that for a given ketone, the magnitude of the shift increases monotonically with the acidity of the alcohol. Spectral shifts increased linearly with decreasing pKa of the donor alcohols. Conversely, when the alcohol remained constant, the spectral shift depended on the C=O bond strength of the ketones. Additionally, very intriguing theoretical research offering a realistic assessment of the distribution of dipole moments in the liquid state [37] can be included in this discussion. Specifically, this paper concluded that the increase in dipole moments of acetone and 2-butanone in the liquid phase compared to the gas phase is not significant due to the lack of hydrogen bonds in the pure ketones themselves. An increase in polarization effects is also anticipated in solutions and mixtures of ketones with proton donors. In this context, it is also possible to suggest that the highest constant obtained in this study for cyclohexanone, as shown in Table 1, is attributed to its relatively rigid structure, stronger polarization, and narrower distribution of dipole moments.

Given the importance of the dipole moments of the carbonyl group and their dynamic character in different solvents, it can also be assumed that the steric and inductive effects are in a dynamic balance that depends on the solvent. Considering the dynamic nature and the multitude of impacts related to the carbonyl group, as well as the indole π-electron condensed system with a strong proton donor group, it seems that the interaction of ketone and indole represents an open challenge for both experimental and theoretical approaches.

### 2.2. Quantum-Chemical Examination of the Interactions

**Figure 3:**
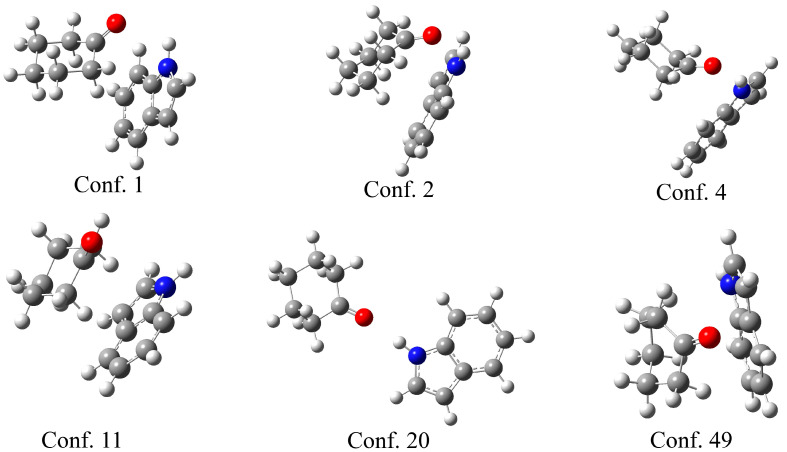
Selected conformers between cyclohexanol and indole, obtained in the CREST program, with the conformer number.

The theoretical support for the previously discussed experimental results was obtained through the optimization of indole–ketone pairs. The CREST (conformer-rotamer ensemble sampling tool) [38] was used to explore the conformational space and identify the initial geometries of the indole–ketone complexes. This program applies a semi-empirical tight-binding quantum chemistry method (GFN2-xTB) [39] to screen the conformational space. During this process, a large number of conformers are located. Upon close inspection of the structures, it is evident that the carbonyl group of the ketone and the amino group of the indole are in close proximity for the most stable conformers. As the complex with cyclohexanone had the highest binding constant, several characteristic examples of the conformers are presented in Figure 3.

As shown in Figure 3, the six selected conformers exhibit distinct geometries governed by different interactions. Three conformers (1, 2, and 4) have the carbonyl group of the ketone directed towards the amino group of the indole, proving that the formation of a hydrogen bond is one of the most important stabilization parameters, as expected from the experimental results. The N-H∙∙∙H distances are 2.20 (conf. 1), 2.43 (conf. 2), and 2.05 Å (conf. 4). Another distinctive feature of these structures is the position of cyclohexanone above the planar structure of indole, which allows for the formation of additional weak interactions between the two ring structures. Once the cyclohexanone carbonyl group is distanced from the amino group, only the weak interactions between rings stabilize the structure, as in the case in conformer 11. Conformer 20 is interesting because the compounds are positioned in a way that only a hydrogen bond is formed, with a bond distance of 2.05 Å. Conformer 49 is included for illustrative purposes, as the distance between the two compounds allows for the formation of only weak interactions between the hydrogen atoms of the ketone and the carbon atoms of the indole. A similar observation was made in complexes with all other ketones.

Although CREST calculations allowed identification of a wide variety of possible conformers, only the geometries were used as starting points for further calculations. No quantitative conclusions were drawn from semi-empirical energy differences. Ten conformers of each pair were then subjected to the MMFF94 (Merck Molecular Force Field)-based calculations [40] in Open Babel [41]. The MMFF94 method demonstrated strong performance and was recommended for hydrogen-bond-donating compounds in a study by Lewis-Atwell and coworkers [42]. The initial search by MMFF94 for the most stable conformers formed between the water molecule and aspirin, paracetamol, and caffeine was presented in the reference [43]. The relative energies of these conformers for each of the pairs are given in Tables S2–S7, together with the optimized geometries in the Supplementary Material. The geometries of these complexes are not identical, but rather very similar, as reflected in their energy values. For example, in the case of acetone, the relative energies range from 0 to 1.6 kJ mol^−1^. With an increase in the complexity of ketones, the relative energies span a much wider range, up to 8.8 kJ mol^−1^ (acetophenone) and 21.2 kJ mol^−1^ (benzophenone). Based on the optimization of the cyclohexanone–indole pair, it can be concluded that the ring structure of cyclohexanone dictates the formation of stabilization interactions, as shown in the previous figure.

The lowest-energy conformers from MMFF94 calculations were selected and optimized at the M06-2X/6-311++G(d,p) level of theory without any geometric constraints. This was completed to ensure that the global minima on the potential energy surface are examined and correlated with the experimental results. The minima on the potential energy surface were proven by the absence of imaginary frequencies. Before exploring the results, it is essential to justify the use of the M06-2X functional for examining hydrogen bonds and other weak interactions formed between ketones and indole. In the paper by Walker and coworkers, it was shown that the M06 functional performed well in predicting general trends in conformer energies and global minima identification of halide ion-amino acid clusters when compared with MP2 [44]. Functional M06-2X was outlined for the optimization of hydrogen-bonded and halogen-bonded complexes by Zhang, Ma, and Wang in reference [45]. Paul examined the red- and blue-shifting hydrogen bonds of cyclic ketones with HF and CHF_3_ and compared the structural and topological parameters obtained by B3LYP, O3LYP, and M06-2X functionals, and the choice of functional proved to be important in characterizing stabilizing effects [46]. Other systems in which intermolecular hydrogen bonds are important for stabilization and changes in spectra were optimized by the M06-2X functional in references [47,48,49].

**Figure 4:**
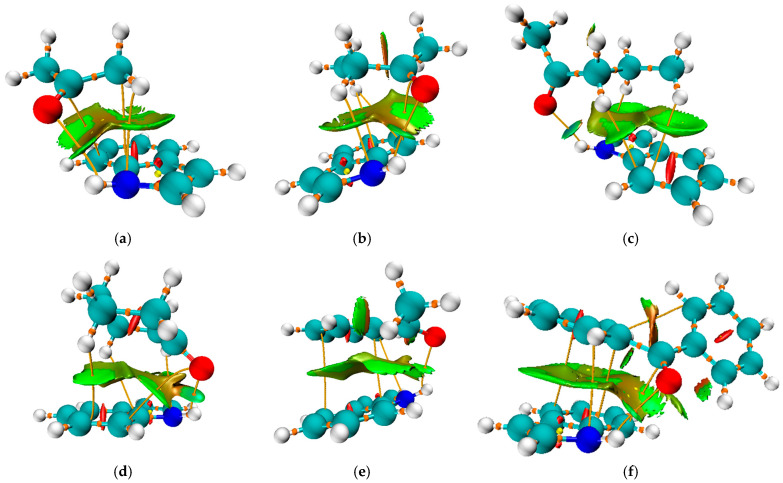
Non-Covalent Interaction (NCI) surface plot of the complexes between indole and (**a**) acetone, (**b**) 2-butanone, (**c**) 2-pentanone, (**d**) cyclohexanone, (**e**) acetophenone, and (**f**) benzophenone, computed based on the reduced density gradient (RDG) at the M06-2X/6-311++G(d,p) level. The isosurface is plotted at RDG = 0.5 a.u. and colored according to the sign(λ_2_)ρ function: blue indicates strong attractive interactions (hydrogen bonding), green indicates van der Waals interactions, and red corresponds to steric repulsion. The surface reveals a π–π interaction region and a hydrogen bond consistent with the geometry optimization results.

This optimization step was taken as an additional proof that the minima are the most stable conformations on the potential energy surface. The optimized structures of the pairs are presented in Figure 4. The binding energy was calculated as the difference between the energy of the complex and the sum of the energies of the separate indole and ketone (Equation (4)), and is listed in Table 2, with the inclusion of the BSSE correction, as explained in Section 3.

Selected structural details and the Quantum theory of Atoms in Molecules (QTAIM) parameters of the important interactions are summarized in Table 2 and Table S1. The total energy density was determined as the sum of kinetic (G(r)) and potential (V(r)) energy densities [50]. Previously, QTAIM analysis has proven helpful in examining inter- and intramolecular hydrogen bonds and predicting their strength [51,52]. The topological properties of hydrogen bonds were suggested by Koch and Popelier [53]. Based on this, the hydrogen bonds should exhibit relatively high electron density, between 0.002 and 0.034 a.u., and Laplacian in the range of 0.024–0.139 a.u.

In the theoretical examination of the binding process, the binding constant is expected to align with the structural and QTAIM parameters of bonds involved in the formation of N-H∙∙∙O. As shown in Table S1, the bond lengths between hydrogen and nitrogen atoms of indole in hydrogen-bonded pairs are in a very narrow range of 1.010 to 1.016 Å (Table S1). The change in the N-H bond length cannot be regarded as a definitive criterion, as the formation of hydrogen bonds leads to significant structural deformation of both the donor and the acceptor, as discussed by Wang and coworkers [54]. This is also reflected in electron density values, with slight differences. The elongation of the C=O bond also depends on the strength of the hydrogen bond and the electron-donating/withdrawing effects of substituents on both sides. The C=O bonds are longer in pairs with higher binding constants, as in pairs with acetone, 2-butanone, 2-pentanone, and cyclohexanone (between 1.229 and 1.234 Å). In complexes with acetophenone and benzophenone, the C=O bond length is shorter (1.214 and 1.218 Å), indicating the presence of other interactions except a hydrogen bond. The electron densities of C=O bonds are inversely proportional to the bond lengths, as expected. The NBO charges on oxygen atoms are similar for all ketones and are between −0.607 and −0.597 *e*, reflecting the bond’s elongation as dictated by the presence of different substituents. It should be noted that the lowest charges on oxygen atoms were found for the ketones with the extended delocalization. A simple correlation between the structural parameters of indole and ketones and the experimental values is not possible, suggesting that the formation of hydrogen bonds is not the sole parameter influencing stability.

On the other hand, the differences in H∙∙∙O bond lengths are much more pronounced, between 2.019 (2-butanone) and 2.384 Å (acetophenone), as shown in Table 2. This result indicates the difference in hydrogen bond strength and orientation. The electron density at the H∙∙∙O BCPs ranges from 0.011 to 0.019 a.u., and the potential energy density spans from −18.4 to −36.3 kJ mol^−1^, both consistent with moderate closed-shell hydrogen bonds. The lowest potential energy density values were obtained for the ketones with the highest binding constant (2-pentanone and cyclohexanone). The total energy densities H(H∙∙∙O) (3.1–6.3 kJ mol^−1^) align with the classification proposed by Popelier [53], confirming weak-to-moderate interaction strength. Acetophenone exhibits the weakest H-bond, correlating with the longest H∙∙∙O distance and lowest ρ(H∙∙∙O). The angle between the hydrogen atom donor and acceptor is also included in Table 2. The highest value of the angle was determined for the 2-butanone–indole pair (140.9°). Other hydrogen bonds have much lower angles, which classify them as weak hydrogen bonds that are distorted due to steric or electronic factors.

The vibrational frequency shifts (Δν) show the highest value for acetophenone (86 cm^−1^), potentially reflecting a significant perturbation of the N-H bond through vibrational coupling or local environment effects, rather than purely H-bonding strength. Benzophenone, despite its lower Δν (23 cm^−1^), has a relatively strong binding energy, suggesting the influence of π–π interactions with indole.

The binding energies (E_b_), calculated with BSSE correction, follow the trend 2-pentanone (47.6 kJ mol^−1^) > cyclohexanone (47.3 kJ mol^−1^) > benzophenone (44.3 kJ mol^−1^) > acetophenone (43.6 kJ mol^−1^) > 2-butanone (41.1 kJ mol^−1^) > acetone (39.8 kJ mol^−1^). These results partially correlate with the experimental binding constants (K_a_), which are highest for cyclohexanone (6.6 M^−1^) and lowest for benzophenone (0.3 M^−1^). The binding energies obtained by DFT methods have to be carefully evaluated [55] and the discrepancies, such as between E_b_ and K_a_ for benzophenone, imply the necessity of examining additional interactions, notably π–π stacking, that influence overall complex stability.

In addition to the previously discussed hydrogen bond, each pair is characterized by further interactions between ketone substituents and the indole, as illustrated in Figure 4. The same figure presents the Non-Covalent Interaction surface plot in which the green area indicates weak interactions. The strength of these interactions was assessed using the approach suggested by Espinosa, in which the interatomic interaction energy is determined as half the value of V(r) [56]. The complex formed between acetone and indole is characterized by the presence of an additional N∙∙∙C bond with an electron density of 0.008 a.u. and an interaction energy of −7.0 kJ mol^−1^. Additionally, there is an interaction between carbon atoms with an energy of −4.0 kJ mol^−1^. When the energies of these interactions are combined, it can be concluded that they significantly influence the stability of the formed complex, in addition to the previously discussed hydrogen bond. In the case of complexes with 2-butanone and 2-pentanone, the interactions between the carbon/hydrogen atom of the ketone and the nitrogen atom of indole are stronger (−7.0 and −7.5 kJ mol^−1^). There are interactions between the hydrogen atom of the ketone and the carbon atom of the indole. The number of these interactions and their strength explain the higher binding energies of 2-butanone and 2-pentanone, at 41.1 and 47.6 kJ mol^−1^, respectively, when compared to acetone (39.8 kJ mol^−1^). The same types of interactions are found in the cyclohexanone–indole pair, with energies of −7.1 (H∙∙∙N), −4.8 (O∙∙∙C), and −5.5/−2.9 kJ mol^−1^ (H∙∙∙C), leading to the binding energy of 47.3 kJ mol^−1^. These interactions also explain the highest binding constant of 6.6 M^−1^.

When acetophenone and benzophenone are considered, an additional type of interaction can be observed, involving the carbon atoms of aromatic moieties. These interactions can be denoted as C∙∙∙C or π−π. In the case of acetophenone, there are two interactions of this type with binding energies of −2.6 and −6.5 kJ mol^−1^. As expected, a higher number of C∙∙∙C interactions was found for the benzophenone–indole pair, with energies of −2.8, −5.9, and −5.2 kJ mol^−1^. The interactions between the nitrogen of indole and the carbon atoms were also observed. This is an additional reason why the experimental shift in N-H band position cannot be used for simple comparison, as several interactions influence the change in the bond strength.

The relationship between binding constant and theoretical parameters was examined by multiple two-parameter linear regression models. In the quantitative assessment of the factors influencing the binding constant K_a_, particular attention was given to DFT, NBO charge, and QTAIM parameters, as presented in Table 2 and Table S1. The multivariate regression analysis confirms that a combination of binding energy E_b_ and oxygen atom partial charge (*q*(*O*)) provides the strongest statistical correlation with the experimentally determined equilibrium constants.

(1) $$K_{a}=-369.63-0.205\cdot E_{b}-634.90\cdot q(O)$$

This model yielded a coefficient of determination (R^2^) value of 0.928, indicating that over 92.8% of the observed variation in K_a_ can be attributed to changes in these two parameters. The average absolute difference between the experimental and calculated value is 0.56 M^−1^. From a chemical perspective, this result aligns with established principles governing the formation of hydrogen bonds. The binding energy *E_b_* reflects the overall stabilization of the complex and integrates contributions from all Non-Covalent Interactions. A more negative *E_b_* correlates with stronger binding and thus larger equilibrium constants. Meanwhile, the partial charge on the oxygen atom *q*(*O*) is a direct indicator of its electronegativity and capacity to act as a hydrogen bond acceptor—a more negative *q*(*O*) enhances the interaction strength with the N-H donor group.

Importantly, these two descriptors remain statistically independent (correlation < 0.5), and together they provide a physically grounded interpretation of the binding process. While *E_b_* captures the energetic consequence of the interaction, *q*(*O*) reflects the electronic predisposition for hydrogen bond formation, making the model both predictive and interpretable.

Nevertheless, limitations must be acknowledged. The dataset comprises only six ketone–indole complexes, which restricts statistical robustness and may exaggerate the apparent precision of the regression. The linearity assumption may also oversimplify the complex interplay of forces involved in hydrogen bonding, especially in solution. Moreover, the current model does not account for solvation effects, entropic contributions, or secondary interactions such as dispersion forces or π-stacking, which could be especially relevant for aromatic ketones. Despite these limitations, the current analysis underscores the value of combining global energetic parameters with localized electronic descriptors in modeling molecular recognition phenomena.

The global reactivity parameters are a set of metrics used to assess the reactivity of compounds based on the energies of the Highest Occupied Molecular Orbital (HOMO) and Lowest Unoccupied Molecular Orbital (LUMO) [57,58,59]. These parameters (Table S3) include ionization potential (IP), vertical electron affinity (EA), chemical potential (μ), electronegativity (χ), chemical hardness (η), and electrophilicity index (ω), calculated as detailed in the Supplementary Material. The HOMO- LUMO gap can be regarded as a measure of the overall reactivity of compounds. The lowest HOMO- LUMO gap values were recorded for benzophenone (7.47 eV) and acetophenone (7.82 eV) due to extensive delocalization within the structure. Acetone, 2-butanone, and 2-pentanone exhibit values for this parameter within a narrow range (between 8.74 and 8.88 eV), as expected from their structural features [60]. Global hardness describes the resistance of the electron cloud to deformation under small perturbations. All the examined compounds possess values for this parameter within 1 eV, ranging from 3.74 (benzophenone) to 4.44 eV (acetone). This parameter is also significantly influenced by delocalization within the structure. Electronegativity, defined by Mulliken, is the average of the ionization potential and electron affinity [61]. Regarding this parameter, the lowest values were observed for cyclohexanone, acetone, 2-butanone, and 2-pentanone. The reactivity of these molecules can be attributed to the absence of steric effects that would hinder reactions with surrounding molecules. The electrophilicity index is another descriptor of reactivity [62]. A higher value of this parameter indicates stronger electrophiles (weaker nucleophiles). Acetophenone and benzophenone are stronger electrophiles, while the other compounds are stronger nucleophiles. Since the interaction with indole involves engagement with positively charged amino groups, the lower reactivity of acetophenone and benzophenone can be understood through this effect. The discussion of the global reactivity parameters demonstrates that the reactivity and overall geometry of the included ketones influence interactions with indole.

To further explore the molecular determinants of hydrogen bond-driven binding affinity, a multiple linear regression analysis was performed using quantum-chemical descriptors derived from frontier molecular orbital theory. Among all tested two-variable combinations, the model incorporating ionization potential (IP) and electrophilicity index (ω) emerged as the most statistically robust, yielding a correlation coefficient of R^2^ = 0.957 and an average absolute error of 0.37 M^−1^. The resulting equation

(2) $$K_{a}=78.224-6.856\cdot \mathrm{IP}-5.77\cdot \omega$$

indicates that both a lower ionization potential and lower electrophilicity favor stronger complex formation. This is chemically intuitive, as a lower IP reflects a more readily polarizable electron cloud, enhancing donor–acceptor interactions, while a lower ω suggests reduced electronic stabilization upon accepting charge density, allowing more effective participation in intermolecular bonding.

The predicted *K_a_* values closely match the experimental measurements for all compounds in the study, including subtle distinctions between structurally similar ketones. For example, cyclohexanone, which exhibits the highest experimental *K_a_* (6.6 M^−1^), was also correctly identified by the model as the strongest binder, with a predicted value of 6.16 M^−1^. On the other end of the spectrum, benzophenone, characterized by high IP and ω values due to the extended aromatic system, exhibited the lowest predicted and experimental affinities (0.27 M^−1^ and 0.3 M^−1^, respectively). These findings reinforce the value of global reactivity descriptors—particularly those derived from HOMO–LUMO characteristics—in capturing the intrinsic electronic features that govern non-covalent binding.

Overall, this model provides a reliable and physically interpretable framework for estimating hydrogen bond strength in structurally diverse ketone systems. Its predictive accuracy surpasses that of previous models based solely on QTAIM or geometric parameters, suggesting that donor–acceptor charge flow, as quantified by frontier orbital theory, plays a central role in modulating intermolecular interaction energies in solution.

## 3. Materials and Methods

### 3.1. FTIR Spectroscopic Measurements

Indole (Merck, Darmstadt, Germany, >99%), carbon tetrachloride (Merck, Darmstadt, Germany, >99.9%), acetone, 2-butanone, 2-pentanone, cyclohexanone (Merck, Darmstadt, Germany, >98%), acetophenone, and benzophenone (Merck, Darmstadt, Germany, >98%) were used without further purification. Structural formulas of selected ketones are shown in Figure 1.

Indole certainly dimerizes in a CCl_4_ solution, which has been proven spectroscopically and theoretically [63,64]. However, dimerization occurs at higher indole concentrations in CCl_4_. In the present study, we used very low indole concentrations at 10^−3^–10^−4^ mol dm^−3^ where dimerization can be neglected. The indole concentration in the carbon tetrachloride solutions was sufficient to observe measurable bands in the IR spectrum, thereby preventing self-association. The concentration of the ketone proton acceptors was varied between 0.01 and 0.3 M. The IR spectra were obtained using a Thermo Scientific Nicolet iS20 instrument. A Deuterated Triglycine Sulfate (DTGS) detector was employed in the IR measurements. The samples were placed in a 1 cm quartz cell, and the spectra were recorded at 298 K. Using Becker’s procedure [33], the hydrogen bonding formation constant can be determined from the difference in monomer band absorbency of pure amide in carbontetrachloride solution and carbon tetrachloride solution in the presence of a proton acceptor.

(3) $$K=\frac{A^{0}-A}{AC_{PA}^{0}}$$

where *A*^0^ is the monomer absorbance for carbon tetrachloride solution containing indole, and *A* is the absorbance of the same band for the solution with both indole and the ketone proton acceptor concentration ($C_{PA}^{0}$). The reported frequencies and half-widths were reproducible to within 0.2 and 1 cm^−1^, respectively. The integrated molar absorption coefficients were determined with an accuracy of ±10%. The equilibrium constants were determined with an average relative standard deviation of 6%.

### 3.2. Theoretical Calculations

The experimental findings were supported by theoretical calculations performed on optimized indole–ketone complexes. Low-energy conformers were generated using the CREST (Conformer–Rotamer Ensemble Sampling Tool) [38], which efficiently explores conformational space through the GFN2-xTB semi-empirical tight-binding method [39]. Ten conformers of each pair were then subjected to the MMFF94 (Merck Molecular Force Field)-based calculations [40] in Open Babel [41]. The structures of indole and six ketones were optimized using the Gaussian 09 Program package [65] at the M06-2X/6-311++G(d,p) level of theory [66,67]. The atomic coordinates of indole–ketone pairs and separate compounds are presented in the Supplementary Information. The number of imaginary frequencies was zero in the individual structures and the interacting pairs. Several starting geometries were selected for each pair, and only the most stable ones are examined in the text. As the shifts in FTIR spectra were monitored in carbon tetrachloride solution, the optimizations of structures were carried out in a simulated solution using the Conductor-like Polarizable Continuum (CPCM) [68] model implemented in Gaussian 09. The discussion of vibrations mainly focuses on the purely N-H stretching vibration, as visualized in GaussView 5 [69]. The stabilization interactions formed between indole and ketones were examined using the Natural Bond Orbital (NBO) charges [70] and Quantum Theory of Atoms in Molecules [71,72], similar to previous work [73]. In the last-mentioned reference, interactions between N-monosubstituted benzamides and three proton acceptors (benzene, acetonitrile, and tetrahydrofuran) were examined by the combination of FTIR spectroscopy, DFT calculation, QTAIM, and NBO analysis. The AIMAll program package [74] was utilized for the QTAIM calculations, starting from the wfx file obtained in Gaussian 09. The Multiwfn program [75,76] was used to prepare a Non-Covalent Interaction (NCI) surface plot that was later visualized in the VMD program [77]. The binding energy was determined as the difference between separate units and their complex, as suggested by Parlak and coworkers [78]:

(4) $$E_{b}=E_{complex}-(E_{indole}+E_{ketone})$$

In the previous equation, *E_complex_*, *E_indole_*, and *E_ketone_* are the electronic energies of the optimized complex formed between indole and ketone, and separate indole and ketone, respectively. The basis set superposition errors (BSSEs) were included in the counterpoise correction method.

### 3.3. Statistical Analysis

To explore the relationship between experimental N-H vibrational redshifts and theoretical descriptors, multiple two-parameter linear regression models were constructed. Each model correlated Δν(NH) values with pairs of quantum-chemical parameters, such as binding energy, hydrogen bond distance, electron density, and energy density at the bond critical point. The regression analysis was performed in Microsoft Excel, and model performance was evaluated based on the coefficient of determination (R^2^) and the average deviation from experimental data.

## 4. Conclusions

This study presents a comprehensive vibrational spectroscopic and quantum-chemical analysis of hydrogen-bonded complexes formed between indole and six ketones of varying structural features. Experimentally determined binding constants (K_a_) from FTIR spectroscopy ranged from 0.3 M^−1^ for benzophenone to 6.6 M^−1^ for cyclohexanone. While alkyl ketones showed consistent interaction patterns, aromatic ketones such as benzophenone exhibited reduced hydrogen bond strength due to steric hindrance and potential π–π interactions, despite their inherent electron-withdrawing character.

CREST conformational sampling followed by MMFF94 and M06-2X/6-311++G(d,p) optimization of the most stable structure revealed that hydrogen bonding is a dominant stabilizing interaction, with N-H∙∙∙O distances ranging from 2.019 Å (2-butanone) to 2.384 Å (acetophenone). Binding energies (Eb), corrected for BSSE, varied between 39.8 kJ mol^−1^ (acetone) and 47.6 kJ mol^−1^ (2-pentanone). QTAIM parameters confirmed closed-shell hydrogen bonding, with electron density at the bond critical points (ρ(H∙∙∙O)) between 0.010–0.019 a.u. and potential energy densities from −18.4 to −36.3 kJ mol^−1^.

Multivariate regression analysis established a strong correlation (R^2^ = 0.928) between K_a_ and the BSSE-corrected binding energy and the NBO charge on the oxygen atom. A second model involving global reactivity parameters—ionization potential (IP) and electrophilicity index (ω)—yielded an even higher correlation (R^2^ = 0.957), with an average absolute error of only 0.37 M^−1^. This model accurately predicted cyclohexanone as the strongest binder and benzophenone as the weakest, in agreement with experiment.

Altogether, the results demonstrate that both local hydrogen bond characteristics and global molecular reactivity govern the strength and geometry of Non-Covalent Interactions, offering a predictive framework for rationalizing complex formation in biologically and chemically relevant systems.

## Figures and Tables

**Table 1:** Spectroscopic parameters for indole–ketone N-H∙∙∙O hydrogen-bonded complexes obtained in IR measurements; Δν (NH) and ν_1/2_ are wavenumber shift and halfwidth of the N-H complex band, respectively; ε and B^0^ are linear and integral molar absorption coefficients, respectively.

Proton Donor	Δν [cm^−1^]	ν_1/2_ [cm^−1^]	ε × 10^−3^ [cm^2^ mol^−1^]	B^0^ × 10^−6^ [cm mol^−1^]	K_a_ ^1^ [M^−1^]
Acetone	72	10	578.8	5.123	4.3
2-Butanone	68	12	434.1	11.32	4.1
2-Pentanone	69	11	394.4	93.42	4.4
Cyclohexanone	77	13	370.1	10.44	6.6
Acetophenone	86	13	3014	127.3	1.1
Benzophenone	23	10	6172	133.7	0.3

^1^ Calculated by Equation (3).

**Table 2:** Selected structural and QTAIM parameters for indole–ketone N-H∙∙∙O hydrogen-bonded complexes.

Proton Donor	E_b_ [kJ mol^−1^]	d(H∙∙∙O) [Å]	ρ(H∙∙∙O) [a.u.]	V(H∙∙∙O) [kJ mol^−1^]	H(H∙∙∙O) [kJ mol^−1^]	Angle (N-H∙∙∙O) [°]
Acetone	39.8	2.060	0.013	−22.6	3.8	137.5
2-Butanone	41.1	2.019	0.010	−18.4	2.7	141.9
2-Pentanone	47.6	2.100	0.019	−36.3	6.3	134.9
Cyclohexanone	47.3	2.244	0.016	−28.8	5.1	119.3
Acetophenone	43.6	2.384	0.011	−19.7	3.1	120.4
Benzophenone	44.3	2.250	0.014	−25.3	4.4	126.2

E_b_—BSSE binding energy, d—bond length, ρ—electron density, V—potential energy density, H—total energy density.

## Data Availability

The data are contained in this article. Further inquiries can be directed to the corresponding author.

## Supplementary Materials

The following supporting information can be downloaded at https://www.mdpi.com/article/10.3390/molecules30132685/s1, Figure S1: Four indole spectra at the concentration range from 0.0001; 0.001; 0.002 and 0.005 M; Table S1: Selected structural and QTAIM parameters for indole–ketone N-H∙∙∙O hydrogen-bonded complexes; Table S2: The relative conformer energies for acetone–indole complex; Table S3: The relative conformer energies for 2-butanone–indole complex; Table S4: The relative conformer energies for 2-pentanone–indole complex; Table S5: The relative conformer energies for cyclohexanone–indole complex; Table S6: The relative conformer energies for acetophenone–indole complex; Table S7: The relative conformer energies for benzophenone–indole complex; Table S8: The global reactivity indexes for the ketones used in the study (in eV); Output .xyz file geometries of indole, six ketones, and their pairs (MMFF94 and M06-2X/6-311++G(d,p)).
