# Supplementary material for: Vibrational Spectroscopic and Quantum-Chemical Study of Indole–Ketone Hydrogen-Bonded Complexes
Source: Molecules. 2025 Jun 21;30(13):2685. doi: 10.3390/molecules30132685 (PMC12251109; doi:10.3390/molecules30132685)
Supplement: Supplementary file 1 [file molecules-30-02685-s001.zip › molecules-3666327-supplementary.pdf]

Vibrational Spectroscopic and Quantum-Chemical Study of Indole–Ketone Hydrogen-Bonded Complexes

Branislav Jović[1], Nataša Negru[1], Dušan Dimić[2,*], Branko Kordić[1]

[1]University of Novi Sad, Faculty of Sciences, TrgDositejaObradovića 3, 21000 Novi Sad, Serbia

[2]University of Belgrade, Faculty of Physical Chemistry, Faculty of Sciences, Studentskitrg 12-16, 11000 Belgrade, Serbia

*Author to whom correspondence should be addressed: (ddimic@ffh.bg.ac.rs)

Supplementary Material

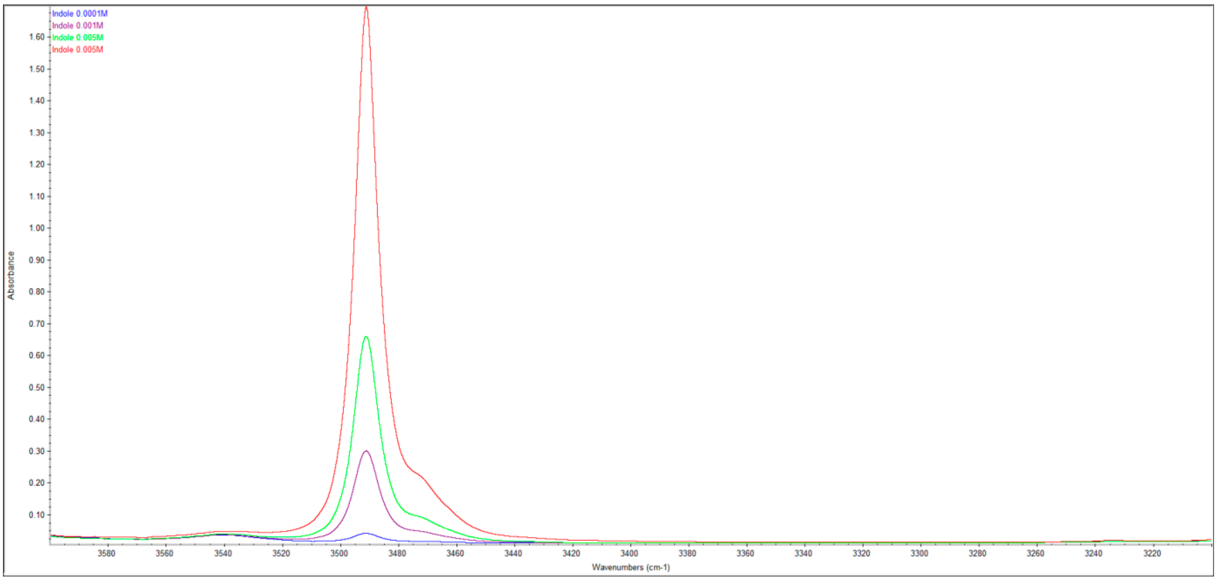

Figure S1 Four indole spectra at the concentration range from 0.0001; 0.001; 0.002 and 0.005 M

From the image S1, free non-bonded N-H indole group can be seen at 3490,97 cm^{-1}, the appearance of the hydrogen bonded indole dimer band at 3472,53) can be clearly seen at concentration of indole at level 0.005 and 0.002 M. In our work, we used very low indole concentrations of 10^{-3}-10^{-4} mol/dm^3 where dimerization can be neglected. It can be seen at figure S1.

Table S1. Selected structural and QTAIM parameters for indole-ketone N-H···O hydrogen-bonded complexes.

Proton donor	d(N−H) [Å]	ρ(N−H) [a.u.]	d(C=O) [Å]	ρ(C=O) [a.u.]	q(O) [*e*]
Acetone	1.016	0.336	1.234	0.402	-0.601
2-Butanone	1.016	0.336	1.229	0.402	-0.603
2-Pentanone	1.016	0.332	1.234	0.400	-0.605
Cyclohexanone	1.014	0.334	1.233	0.400	-0.607
Acetophenone	1.010	0.336	1.214	0.400	-0.597
Benzophenone	1.010	0.335	1.218	0.397	-0.598
E_b – BSSE binding energy, d – bond length, ρ – electron density, q – NBO charge					

Table S2. The relative conformer energies for acetone-indole complex.

Conformer	Relative energy [kJ mol^{-1}]
1	1.6
2	1.2
3	1.9
4	0.9
5	1.1
6	0.9
7	0.7
8	1.7
**9**	**0.0**
10	1.6

Table S3. The relative conformer energies for 2-butanone-indole complex.

Conformer	Relative energy [kJ mol^{-1}]
1	4.5
2	4.0
3	4.9
4	6.0
5	2.1
6	3.5
7	0.3
8	0.6
9	2.5
**10**	**0.0**

Table S4. The relative conformer energies for 2-pentanone-indole complex.

Conformer	Relative energy [kJ mol^{-1}]
1	0.1

**2**	**0.0**
3	0.1
4	3.7
5	2.6
6	3.2
7	0.7
8	3.6
9	3.5
10	6.0

Table S5. The relative conformer energies for cyclohexanone-indole complex.

Conformer	Relative energy [kJ mol^{-1}]
**1**	**0.0**
2	9.1
3	3.5
4	2.5
5	1.9
6	2.2
7	1.5
8	4.1
9	6.8
10	5.2

Table S6. The relative conformer energies for acetophenone-indole complex.

Conformer	Relative energy [kJ mol^{-1}]
1	2.5
2	5.3
3	5.0
4	8.8
5	2.8
6	5.3
7	6.3
8	2.6
**9**	**0.0**
10	0.2

Table S7. The relative conformer energies for benzophenone-indole complex.

Conformer	Relative energy [kJ mol^{-1}]
1	21.2
**2**	**0.0**
3	1.0
4	12.4
5	0.5

6	1.1
7	0.1
8	6.6
9	3.3
10	11.2

$$Ionization\ potential\ (IP) = -E_{HOMO} \quad (1)$$
$$Electron\ Affinity\ (EA) = -E_{LUMO} \quad (2)$$
$$Hardness\ (\eta) \approx (IP - EA)/2 \quad (3)$$
$$Electronegativity\ (\chi) \approx (IP + EA)/2) \quad (4)$$
$$Chemical\ potential\ (\mu) \approx -\chi \quad (5)$$
$$Electrophilicity\ index\ (\omega) \approx \mu^2/2\eta \quad (6)$$

Table S8. The global reactivity indexes for the ketones used in the study (in eV)

Compound	HOMO-LUMO gap	IP	EA	η	χ	μ	ω
Acetone	8.88	8.95	0.07	4.44	4.51	-4.51	2.29
2-Butanone	8.74	8.82	0.09	4.37	4.45	-4.45	2.27
2-Pentanone	8.74	8.80	0.06	4.37	4.43	-4.43	2.25
Cyclohexanone	8.45	8.60	0.15	4.23	4.38	-4.38	2.27
Acetophenone	7.82	8.73	0.91	3.91	4.82	-4.82	2.98
Benzophenone	7.47	8.65	1.18	3.74	4.91	-4.91	3.23

Output file geometries of separate compounds:

Indole

C 0.2480151 0.7472642 -0.0000788
C 0.2484781 -0.6682038 0.0000009
C -0.9331891 -1.4157444 0.0000823
C -2.1279909 -0.7181640 0.0000561
C -2.1528356 0.6910495 -0.0000151
C -0.9813939 1.4258969 -0.0000759
C 1.6233024 1.1647419 0.0000700
C 2.3815038 0.0291039 -0.0000644
H -0.9139656 -2.4994191 0.0001418
H -3.0631680 -1.2655174 0.0000960
H -3.1082265 1.2021487 -0.0000009
H -1.0085966 2.5096911 -0.0000928
H 1.9971090 2.1762169 0.0001212
H 3.4538876 -0.0876248 -0.0000905
N 1.5615834 -1.0768597 0.0000457

H 1.8765372 -2.0331431 -0.0003460

Acetone

O -0.0001220 1.3934590 -0.0000030
C -0.0000020 0.1845970 0.0000150
C 1.2834950 -0.6114220 0.0021880
H 2.1372080 0.0508000 0.1301460
H 1.2629640 -1.3615890 0.7958820
H 1.3735820 -1.1475220 -0.9465500
C -1.2834050 -0.6115890 -0.0021800
H -1.3733250 -1.1478790 0.9464570
H -2.1372480 0.0505070 -0.1299540
H -1.2627350 -1.3615120 -0.7960980

2-butanone

O -0.9719830 1.3129890 -0.2151090
C -0.5597700 0.2214520 0.1042300
C 0.8242690 0.0452510 0.6935640
H 1.0839330 0.9576150 1.2322060
H 0.8275940 -0.7944570 1.3929160
C -1.3795490 -1.0304740 -0.0938890
H -1.7078750 -1.3894890 0.8859800
H -2.2487130 -0.8225390 -0.7145350
H -0.7726090 -1.8220350 -0.5391270
C 1.8350490 -0.2073540 -0.4348510
H 1.8466710 0.6341510 -1.1294450
H 2.8385810 -0.3288230 -0.0259990
H 1.5882910 -1.1115830 -0.9954460

2-pentanone

O 1.6387860 -1.2652150 -0.1740950
C 1.1488570 -0.1961290 0.1104160
C -0.2592640 -0.0987720 0.6569500
H -0.4680380 -1.0001750 1.2373630
H -0.3525980 0.7741480 1.3098190
C 1.8912120 1.1018850 -0.0950400
H 2.1534170 1.5127470 0.8843370
H 2.7968000 0.9356800 -0.6748610
H 1.2500730 1.8353990 -0.5894640
C -1.2648480 0.0139690 -0.5005410
H -1.1544110 -0.8622600 -1.1450930
H -1.0261600 0.8902590 -1.1114240
C -2.7007090 0.1149590 0.0062470
H -3.4066580 0.1861270 -0.8231060
H -2.9641590 -0.7635830 0.5999730
H -2.8300430 0.9979050 0.6370250

Cyclohexanone

O -2.2691140 -0.0000900 -0.3739470
C -1.1457740 -0.0000530 0.0756310
C -0.3898240 1.2784300 0.3696730
C -0.3896660 -1.2784940 0.3694440
C 0.9989070 1.2589590 -0.2905420
H -0.2658930 1.3424590 1.4576900
H -0.9911790 2.1266100 0.0418690
C 0.9992330 -1.2589000 -0.2903020
H -0.2662480 -1.3427310 1.4575250
H -0.9908110 -2.1267090 0.0413210
C 1.7767730 0.0001600 0.0951760
H 1.5495330 2.1575960 -0.0044880
H 0.8787260 1.2905430 -1.3788280
H 1.5499520 -2.1573420 -0.0038030
H 0.8793880 -1.2908670 -1.3786120
H 2.7545710 0.0002250 -0.3923460
H 1.9569850 0.0003210 1.1767710

Acetophenone

O 2.2082560 -1.3032740 0.0003150
C 1.6966650 -0.2051490 0.0000550
C 2.5410250 1.0468250 -0.0002790
H 2.3231610 1.6532330 -0.8824010
H 2.3233160 1.6536440 0.8815950
H 3.5910220 0.7636940 -0.0002950
C 0.2024790 -0.0620060 0.0000160
C -0.4215730 1.1871700 0.0001460
C -0.5782290 -1.2210870 -0.0001350
C -1.8100550 1.2759080 0.0001350
H 0.1680330 2.0960100 0.0002470
C -1.9628780 -1.1323070 -0.0001590
H -0.0796770 -2.1827930 -0.0002530
C -2.5803720 0.1175560 -0.0000050
H -2.2888740 2.2475920 0.0002330
H -2.5635820 -2.0338760 -0.0002850
H -3.6618240 0.1872230 -0.0000160

Benzophenone

O -0.0000420 2.3256610 0.0000110
C -0.0000110 1.1119940 -0.0000090
C -1.2937780 0.3574800 0.0303960
C -1.4160130 -0.8785660 0.6688330
C -2.4190000 0.9551280 -0.5427460
C -2.6536740 -1.5107030 0.7296450
H -0.5518880 -1.3354060 1.1368120
C -3.6483540 0.3119780 -0.5006420
H -2.3117590 1.9223570 -1.0191930
C -3.7665950 -0.9212110 0.1378310
H -2.7486410 -2.4619970 1.2390480

H	-4.5157500	0.7714870	-0.9589670
H	-4.7278070	-1.4200280	0.1775520
C	1.2937850	0.3574970	-0.0304240
C	2.4190100	0.9551560	0.5427330
C	1.4160270	-0.8785450	-0.6688280
C	3.6483420	0.3119920	0.5006660
H	2.3117510	1.9224000	1.0191420
C	2.6537110	-1.5106830	-0.7296600
H	0.5519180	-1.3354050	-1.1368130
C	3.7666000	-0.9212150	-0.1378130
H	4.5157330	0.7714680	0.9590330
H	2.7486600	-2.4619630	-1.2390940
H	4.7278180	-1.4200230	-0.1774900

Output file geometries of the three most stable conformers:

Indole-acetone

Conformer 1

C	-1.7124	-0.4201	-0.2992
C	-0.7538	-0.2212	0.6939
C	-0.4359	1.0460	1.1970
C	-1.1072	2.1445	0.6533
C	-2.0670	1.9771	-0.3428
C	-2.3842	0.7007	-0.8236
C	-1.7572	-1.8170	-0.5633
C	-0.8212	-2.4269	0.2408
H	0.3210	1.1749	1.9646
H	-0.8732	3.1442	1.0123
H	-2.5769	2.8456	-0.7524
H	-3.1319	0.5792	-1.6014
H	-2.3908	-2.3178	-1.2831
H	-0.5188	-3.4614	0.3313
N	-0.2174	-1.4546	0.9947
O	2.6935	-0.6537	0.6234
H	0.5661	-1.5980	1.6208
C	2.4979	0.0786	-0.3465
C	2.8204	1.5486	-0.3028
H	3.6118	1.7687	-1.0214
H	1.9264	2.1303	-0.5407
H	3.1620	1.8211	0.6994
C	1.9157	-0.4448	-1.6334
H	2.5790	-0.1967	-2.4638
H	1.8079	-1.5297	-1.5759
H	0.9337	0.0055	-1.8004

Conformer 2

C	-2.0602	-0.4907	-0.1555

C	-0.8426	-0.1528	0.4336
C	-0.4839	1.1686	0.7267
C	-1.3942	2.1752	0.3946
C	-2.6199	1.8679	-0.1933
C	-2.9678	0.5388	-0.4706
C	-2.0764	-1.9045	-0.3028
C	-0.8806	-2.3906	0.1777
H	0.4685	1.4053	1.1885
H	-1.1433	3.2120	0.6018
H	-3.3160	2.6647	-0.4378
H	-3.9254	0.3085	-0.9254
H	-2.8748	-2.5004	-0.7241
H	-0.4889	-3.3959	0.2386
N	-0.1426	-1.3249	0.6235
O	2.6791	-0.6793	0.7089
H	0.8037	-1.3727	0.9912
C	2.9423	0.0820	-0.2207
C	3.9993	1.1493	-0.1038
H	4.8438	0.8949	-0.7483
H	3.5785	2.1136	-0.4003
H	4.3433	1.2209	0.9312
C	2.1865	0.0279	-1.5224
H	1.7950	-0.9812	-1.6785
H	1.3666	0.7477	-1.4959
H	2.8539	0.2702	-2.3549

Conformer 3

C	-2.0491	-0.3473	0.4865
C	-0.8922	-0.2046	-0.2786
C	-0.5424	0.9980	-0.9049
C	-1.3960	2.0897	-0.7310
C	-2.5600	1.9758	0.0268
C	-2.9012	0.7631	0.6377
C	-2.0742	-1.6873	0.9588
C	-0.9454	-2.3198	0.4918
H	0.3629	1.0828	-1.4971
H	-1.1492	3.0404	-1.1979
H	-3.2131	2.8366	0.1442
H	-3.8116	0.6824	1.2237
H	-2.8379	-2.1376	1.5820
H	-0.5818	-3.3297	0.6266
N	-0.2364	-1.4173	-0.2558
O	2.5793	-0.9590	-0.8075
H	0.6665	-1.5868	-0.6913
C	2.9703	-0.0117	-0.1281
C	2.3523	0.3095	1.2049
H	1.5545	1.0424	1.0654
H	1.9486	-0.6031	1.6531
H	3.1052	0.7204	1.8796
C	4.0559	0.9194	-0.6013

H	4.2870	0.7222	-1.6517
H	3.7138	1.9547	-0.5062
H	4.9533	0.7657	0.0026

Conformer 4

C	-2.0925	-0.4027	-0.1298
C	-0.8488	-0.1404	0.4391
C	-0.4352	1.1499	0.7910
C	-1.3175	2.2040	0.5405
C	-2.5691	1.9712	-0.0257
C	-2.9721	0.6728	-0.3616
C	-2.1645	-1.8073	-0.3524
C	-0.9740	-2.3584	0.0636
H	0.5380	1.3285	1.2359
H	-1.0234	3.2194	0.7953
H	-3.2436	2.8048	-0.2066
H	-3.9497	0.5000	-0.8019
H	-2.9954	-2.3505	-0.7810
H	-0.6165	-3.3787	0.0582
N	-0.1855	-1.3451	0.5445
O	2.6572	-0.8199	0.6033
H	0.7676	-1.4453	0.8821
C	2.9571	-0.0200	-0.2822
C	2.1727	0.0629	-1.5659
H	2.8354	0.3162	-2.3965
H	1.7110	-0.9049	-1.7762
H	1.4026	0.8308	-1.4698
C	4.0909	0.9585	-0.1232
H	4.4606	0.9340	0.9055
H	4.9019	0.6887	-0.8042
H	3.7363	1.9688	-0.3436

Conformer 5

C	-2.0758	-0.4943	-0.1469
C	-0.8514	-0.1488	0.4237
C	-0.4958	1.1755	0.7069
C	-1.4152	2.1767	0.3835
C	-2.6470	1.8613	-0.1859
C	-2.9921	0.5305	-0.4524
C	-2.0867	-1.9094	-0.2881
C	-0.8827	-2.3873	0.1776
H	0.4622	1.4188	1.1546
H	-1.1658	3.2162	0.5834
H	-3.3511	2.6548	-0.4235
H	-3.9556	0.2930	-0.8931
H	-2.8888	-2.5103	-0.6953
H	-0.4854	-3.3919	0.2376
N	-0.1436	-1.3170	0.6086
O	2.6831	-0.6770	0.6994

H	0.8075	-1.3594	0.9662
C	2.9683	0.0841	-0.2238
C	4.0627	1.1086	-0.0999
H	3.6777	2.0910	-0.3836
H	4.4120	1.1547	0.9344
H	4.8963	0.8292	-0.7491
C	2.2026	0.0689	-1.5197
H	1.7878	-0.9279	-1.6897
H	1.4000	0.8059	-1.4754
H	2.8689	0.3098	-2.3523

Conformer 6

C	-2.0998	-0.4004	-0.1270
C	-0.8546	-0.1339	0.4406
C	-0.4467	1.1568	0.7970
C	-1.3323	2.2078	0.5500
C	-2.5840	1.9725	-0.0149
C	-2.9822	0.6734	-0.3546
C	-2.1646	-1.8031	-0.3551
C	-0.9724	-2.3519	0.0589
H	0.5293	1.3372	1.2382
H	-1.0404	3.2234	0.8058
H	-3.2603	2.8049	-0.1955
H	-3.9589	0.4981	-0.7951
H	-2.9944	-2.3485	-0.7842
H	-0.6113	-3.3712	0.0503
N	-0.1871	-1.3368	0.5410
O	2.6581	-0.8207	0.6038
H	0.7681	-1.4351	0.8755
C	2.9708	-0.0256	-0.2864
C	2.1718	0.0807	-1.5566
H	1.4163	0.8604	-1.4417
H	2.8272	0.3317	-2.3935
H	1.6909	-0.8773	-1.7713
C	4.1348	0.9134	-0.1388
H	3.8080	1.9350	-0.3538
H	4.5193	0.8790	0.8832
H	4.9277	0.6219	-0.8320

Conformer 7

C	-2.0282	-0.4845	0.2015
C	-0.8334	-0.1634	-0.4419
C	-0.4680	1.1539	-0.7455
C	-1.3445	2.1736	-0.3664
C	-2.5460	1.8827	0.2769
C	-2.9024	0.5586	0.5625
C	-2.0595	-1.8993	0.3433
C	-0.8947	-2.4011	-0.1904
H	0.4658	1.3776	-1.2512

H	-1.0858	3.2078	-0.5800
H	-3.2168	2.6901	0.5580
H	-3.8416	0.3410	1.0614
H	-2.8474	-2.4842	0.7987
H	-0.5200	-3.4124	-0.2705
N	-0.1603	-1.3453	-0.6655
O	2.6572	-0.6835	-0.7589
H	0.7697	-1.4045	-1.0700
C	2.8934	0.0878	0.1758
C	3.8443	1.2421	0.0306
H	4.7323	1.0599	0.6410
H	4.1430	1.3496	-1.0159
H	3.3514	2.1645	0.3495
C	2.1993	-0.0575	1.5052
H	1.8630	-1.0896	1.6363
H	2.8910	0.1857	2.3143
H	1.3439	0.6185	1.5420

Conformer 8

C	-2.0203	-0.4802	-0.1715
C	-0.8142	-0.1707	0.4548
C	-0.4363	1.1422	0.7622
C	-1.3144	2.1688	0.4059
C	-2.5275	1.8890	-0.2197
C	-2.8953	0.5696	-0.5103
C	-2.0621	-1.8933	-0.3253
C	-0.8913	-2.4055	0.1864
H	0.5077	1.3579	1.2524
H	-1.0471	3.2003	0.6233
H	-3.1996	2.7022	-0.4831
H	-3.8433	0.3594	-0.9958
H	-2.8606	-2.4694	-0.7726
H	-0.5221	-3.4200	0.2523
N	-0.1430	-1.3583	0.6566
O	2.6858	-0.6950	0.7073
H	0.7941	-1.4258	1.0432
C	2.8752	0.0894	-0.2268
C	3.7988	1.2675	-0.1048
H	4.1141	1.3896	0.9344
H	4.6777	1.1052	-0.7325
H	3.2754	2.1748	-0.4196
C	2.1468	-0.0599	-1.5376
H	2.8064	0.2159	-2.3630
H	1.8371	-1.1001	-1.6724
H	1.2714	0.5927	-1.5410

Conformer 9

C	-2.0163	-0.4781	-0.1696
C	-0.8199	-0.1717	0.4776

C	-0.4458	1.1394	0.7961
C	-1.3160	2.1688	0.4278
C	-2.5191	1.8931	-0.2187
C	-2.8842	0.5744	-0.5192
C	-2.0557	-1.8913	-0.3275
C	-0.8931	-2.4061	0.1999
H	0.4891	1.3513	1.3045
H	-1.0502	3.1990	0.6526
H	-3.1846	2.7084	-0.4913
H	-3.8252	0.3683	-1.0198
H	-2.8466	-2.4664	-0.7899
H	-0.5236	-3.4205	0.2659
N	-0.1535	-1.3602	0.6872
O	2.6604	-0.6900	0.6875
H	0.7786	-1.4286	1.0862
C	2.8546	0.0915	-0.2479
C	3.7550	1.2868	-0.1049
H	3.2138	2.1891	-0.4009
H	4.0691	1.3930	0.9365
H	4.6374	1.1556	-0.7349
C	2.1632	-0.0837	-1.5726
H	1.2920	0.5742	-1.6185
H	2.8503	0.1581	-2.3864
H	1.8466	-1.1235	-1.6869

Conformer 10

C	-1.7124	-0.4201	-0.2992
C	-0.7538	-0.2212	0.6939
C	-0.4359	1.0460	1.1970
C	-1.1072	2.1445	0.6533
C	-2.0670	1.9771	-0.3428
C	-2.3842	0.7007	-0.8236
C	-1.7572	-1.8170	-0.5633
C	-0.8212	-2.4269	0.2408
H	0.3210	1.1749	1.9646
H	-0.8732	3.1442	1.0123
H	-2.5769	2.8456	-0.7524
H	-3.1319	0.5792	-1.6014
H	-2.3908	-2.3178	-1.2831
H	-0.5188	-3.4614	0.3313
N	-0.2174	-1.4546	0.9947
O	2.6935	-0.6537	0.6234
H	0.5661	-1.5980	1.6208
C	2.4979	0.0786	-0.3465
C	2.8204	1.5486	-0.3028
H	3.6118	1.7687	-1.0214
H	1.9264	2.1303	-0.5407
H	3.1620	1.8211	0.6994
C	1.9157	-0.4448	-1.6334
H	2.5790	-0.1967	-2.4638

H	1.8079	-1.5297	-1.5759
H	0.9337	0.0055	-1.8004

Indole-2-butanone

Conformer 1

C	1.8669	-0.3879	-0.2849
C	0.9355	-0.2888	0.7479
C	0.6041	0.9283	1.3559
C	1.2334	2.0817	0.8802
C	2.1678	2.0141	-0.1514
C	2.4988	0.7861	-0.7386
C	1.9277	-1.7579	-0.6631
C	1.0333	-2.4506	0.1211
H	-0.1345	0.9790	2.1500
H	0.9845	3.0452	1.3189
H	2.6430	2.9240	-0.5093
H	3.2212	0.7436	-1.5478
H	2.5435	-2.1851	-1.4431
H	0.7537	-3.4952	0.1384
N	0.4351	-1.5538	0.9676
O	-2.4382	-0.8437	1.0194
H	-0.3316	-1.7598	1.5981
C	-2.4560	-0.0402	0.0845
C	-2.1171	-0.4738	-1.3309
H	-1.5174	-1.3886	-1.2699
H	-3.0532	-0.7210	-1.8403
C	-2.8903	1.3871	0.3044
H	-2.0329	2.0574	0.2140
H	-3.6578	1.6566	-0.4243
H	-3.3132	1.4902	1.3078
C	-1.3520	0.5659	-2.1283
H	-1.9661	1.4511	-2.3210
H	-0.4586	0.9032	-1.5992
H	-1.0466	0.1549	-3.0958

Conformer 2

C	1.6833	-0.0857	0.7584
C	1.0621	-0.6539	-0.3529
C	1.0013	-0.0153	-1.5972
C	1.5937	1.2461	-1.7056
C	2.2219	1.8399	-0.6122
C	2.2754	1.1851	0.6250
C	1.5347	-1.0033	1.8352
C	0.8260	-2.0872	1.3689
H	0.4970	-0.4725	-2.4429
H	1.5546	1.7736	-2.6559
H	2.6694	2.8251	-0.7167
H	2.7591	1.6584	1.4739

H	1.8939	-0.8738	2.8474
H	0.4838	-2.9868	1.8626
N	0.5428	-1.8662	0.0462
O	-2.3029	-1.2941	-0.8097
H	-0.0294	-2.4605	-0.5412
C	-2.3566	-0.2687	-0.1222
C	-2.4712	1.0952	-0.7755
H	-2.0501	1.0229	-1.7827
H	-3.5340	1.3340	-0.8672
C	-2.3926	-0.3520	1.3801
H	-2.4881	-1.3968	1.6876
H	-1.4696	0.0549	1.8010
H	-3.2567	0.2001	1.7615
C	-1.7577	2.2038	-0.0201
H	-2.2279	2.3918	0.9507
H	-0.7121	1.9519	0.1657
H	-1.7914	3.1354	-0.5945

Conformer 3

C	1.7916	-0.3174	-0.2965
C	0.9440	-0.3078	0.8111
C	0.6283	0.8619	1.5130
C	1.1804	2.0611	1.0537
C	2.0241	2.0834	-0.0548
C	2.3421	0.9020	-0.7360
C	1.8731	-1.6628	-0.7503
C	1.0677	-2.4295	0.0614
H	-0.0451	0.8444	2.3640
H	0.9417	2.9898	1.5667
H	2.4411	3.0273	-0.3972
H	2.9959	0.9282	-1.6023
H	2.4351	-2.0247	-1.6005
H	0.8187	-3.4817	0.0368
N	0.4959	-1.5986	0.9899
O	-2.4109	-0.7743	0.5997
H	-0.2160	-1.8682	1.6564
C	-2.3182	0.2902	-0.0079
C	-1.7955	0.4005	-1.4301
H	-2.4681	1.0590	-1.9905
H	-0.8084	0.8728	-1.4047
C	-2.7327	1.5885	0.6403
H	-2.8722	1.4383	1.7139
H	-1.9575	2.3439	0.4926
H	-3.6721	1.9277	0.1954
C	-1.7177	-0.9380	-2.1388
H	-2.7065	-1.4020	-2.2008
H	-1.3299	-0.8095	-3.1544
H	-1.0620	-1.6347	-1.6126

Conformer 4

C	1.8018	-0.4319	0.2948
C	1.0478	0.0464	-0.7765
C	0.7841	1.4078	-0.9684
C	1.2881	2.3037	-0.0212
C	2.0325	1.8536	1.0673
C	2.3022	0.4898	1.2345
C	1.8704	-1.8461	0.1604
C	1.1488	-2.1965	-0.9580
H	0.1961	1.7556	-1.8117
H	1.0937	3.3676	-0.1360
H	2.4127	2.5671	1.7942
H	2.8858	0.1468	2.0833
H	2.3778	-2.5341	0.8231
H	0.9290	-3.1606	-1.3958
N	0.6375	-1.0485	-1.5050
O	-2.4410	-0.4001	-1.0643
H	0.0048	-1.0067	-2.2938
C	-2.3860	0.2949	-0.0499
C	-2.0110	-0.2551	1.3152
H	-1.0268	0.1399	1.5853
H	-2.7397	0.1131	2.0445
C	-2.7039	1.7673	-0.1038
H	-3.6747	1.9458	0.3644
H	-1.9287	2.3344	0.4168
H	-2.7372	2.0992	-1.1452
C	-1.9928	-1.7709	1.3566
H	-1.6877	-2.1210	2.3476
H	-2.9845	-2.1779	1.1377
H	-1.2976	-2.1850	0.6218

Conformer 5

C	1.8325	-0.1557	0.7595
C	1.1059	-0.6495	-0.3229
C	0.9430	0.0631	-1.5164
C	1.5385	1.3249	-1.6009
C	2.2621	1.8499	-0.5317
C	2.4196	1.1197	0.6528
C	1.7847	-1.1437	1.7816
C	1.0256	-2.1935	1.3161
H	0.3675	-0.3424	-2.3425
H	1.4268	1.9073	-2.5125
H	2.7109	2.8364	-0.6165
H	2.9863	1.5351	1.4805
H	2.2470	-1.0848	2.7578
H	0.7309	-3.1259	1.7783
N	0.6117	-1.8818	0.0472
O	-2.2666	-1.3388	-0.6295

H	-0.0197	-2.4341	-0.5194
C	-2.3917	-0.2412	-0.0786
C	-2.8478	0.9729	-0.8639
H	-2.6397	0.7829	-1.9234
H	-3.9334	1.0650	-0.7442
C	-2.1792	-0.0922	1.4065
H	-1.2868	0.5086	1.5975
H	-3.0554	0.3764	1.8600
H	-2.0421	-1.0790	1.8580
C	-2.1658	2.2641	-0.4525
H	-1.0777	2.1654	-0.4790
H	-2.4527	3.0769	-1.1267
H	-2.4481	2.5568	0.5636

Conformer 6

C	1.8219	-0.4707	0.2300
C	0.9637	-0.2403	0.8449
C	0.6968	1.0412	1.3405
C	1.3255	2.1206	0.7134
C	2.1839	1.9219	-0.3666
C	2.4421	0.6321	-0.8475
C	1.8433	-1.8752	-0.4544
C	0.9973	-2.4601	0.4610
H	0.0138	1.1947	2.1706
H	1.1317	3.1302	1.0680
H	2.6552	2.7765	-0.8455
H	3.1090	0.4864	-1.6919
H	2.4054	-2.3985	-1.2163
H	0.7143	-3.4917	0.6185
N	0.4675	-1.4636	1.2387
O	-2.4574	-0.7092	1.0063
H	-0.2421	-1.5872	1.9512
C	-2.3552	-0.2647	-0.1417
C	-2.6656	1.1879	-0.4429
H	-3.7229	1.2551	-0.7199
H	-2.5200	1.7586	0.4812
C	-2.0032	-1.1772	-1.2897
H	-1.0160	-0.9207	-1.6804
H	-1.9877	-2.2124	-0.9444
H	-2.7560	-1.0861	-2.0784
C	-1.8048	1.7936	-1.5357
H	-2.0066	1.3293	-2.5062
H	-2.0064	2.8652	-1.6288
H	-0.7415	1.6632	-1.3244

Conformer 7

C	1.5049	-0.1281	0.9201
C	1.1988	-0.6259	-0.3468

C	1.3043	0.1467	-1.5084
C	1.7189	1.4743	-1.3668
C	2.0182	2.0031	-0.1127
C	1.9193	1.2122	1.0386
C	1.2944	-1.1864	1.8470
C	0.8503	-2.2818	1.1408
H	1.0577	-0.2605	-2.4837
H	1.8016	2.1038	-2.2500
H	2.3322	3.0403	-0.0256
H	2.1556	1.6312	2.0118
H	1.4387	-1.1443	2.9191
H	0.5613	-3.2725	1.4627
N	0.7835	-1.9287	-0.1816
O	-2.3754	-1.0346	-0.6791
H	0.4468	-2.5211	-0.9284
C	-2.2639	-0.0952	0.1096
C	-2.1228	1.3479	-0.3400
H	-2.9098	1.9351	0.1447
H	-1.1544	1.7227	0.0037
C	-2.3104	-0.3204	1.5996
H	-2.1759	-1.3835	1.8176
H	-1.5079	0.2391	2.0866
H	-3.2781	0.0081	1.9853
C	-2.2342	1.5065	-1.8452
H	-3.2200	1.1854	-2.1984
H	-2.0935	2.5541	-2.1291
H	-1.4841	0.9069	-2.3679

Conformer 8

C	1.6662	-0.5202	-0.2741
C	1.0151	-0.2188	0.9210
C	0.8845	1.0878	1.4048
C	1.4218	2.1199	0.6301
C	2.0669	1.8512	-0.5755
C	2.1997	0.5358	-1.0374
C	1.6107	-1.9321	-0.4370
C	0.9192	-2.4516	0.6335
H	0.3679	1.2993	2.3358
H	1.3259	3.1482	0.9717
H	2.4707	2.6702	-1.1655
H	2.7036	0.3347	-1.9777
H	2.0194	-2.5037	-1.2590
H	0.6396	-3.4663	0.8833
N	0.5538	-1.4083	1.4444
O	-2.5292	-0.5966	0.5781
H	-0.0061	-1.4861	2.2822
C	-2.1601	-0.3186	-0.5636
C	-2.0012	1.1088	-1.0552
H	-0.9473	1.2726	-1.2972

H	-2.5845	1.2210	-1.9752
C	-1.8791	-1.4013	-1.5742
H	-0.9411	-1.1909	-2.0947
H	-1.7894	-2.3658	-1.0683
H	-2.6998	-1.4452	-2.2941
C	-2.4644	2.1364	-0.0393
H	-1.9258	2.0347	0.9078
H	-2.2982	3.1490	-0.4196
H	-3.5323	2.0198	0.1723

Conformer 9

C	1.4329	-0.1294	0.9409
C	1.2282	-0.6172	-0.3505
C	1.3864	0.1767	-1.4921
C	1.7432	1.5153	-1.3056
C	1.9405	2.0336	-0.0260
C	1.7930	1.2225	1.1046
C	1.1892	-1.2059	1.8383
C	0.8216	-2.3012	1.0898
H	1.2155	-0.2227	-2.4868
H	1.8589	2.1613	-2.1716
H	2.2082	3.0800	0.0953
H	1.9460	1.6338	2.0970
H	1.2536	-1.1748	2.9186
H	0.5294	-3.3009	1.3784
N	0.8402	-1.9321	-0.2290
O	-2.3104	-1.0942	-0.5818
H	0.5648	-2.5223	-1.0051
C	-2.2169	-0.0870	0.1219
C	-2.0874	1.3106	-0.4561
H	-2.8860	1.9300	-0.0359
H	-1.1282	1.7256	-0.1372
C	-2.2885	-0.1770	1.6235
H	-2.1105	-1.2080	1.9423
H	-1.5243	0.4622	2.0727
H	-3.2790	0.1398	1.9581
C	-2.1806	1.3280	-1.9711
H	-3.1568	0.9621	-2.3038
H	-2.0497	2.3475	-2.3482
H	-1.4154	0.6939	-2.4273

Conformer 10

C	2.3900	-0.4700	0.0510
C	1.0850	-0.2092	0.4694
C	0.5916	1.0874	0.6493
C	1.4591	2.1485	0.3789
C	2.7700	1.9165	-0.0381
C	3.2502	0.6134	-0.2033

C	2.5328	-1.8799	-0.0185
C	1.3277	-2.4428	0.3378
H	-0.4264	1.2657	0.9766
H	1.1091	3.1688	0.5019
H	3.4274	2.7577	-0.2350
H	4.2726	0.4415	-0.5244
H	3.4222	-2.4248	-0.3066
H	1.0119	-3.4738	0.4057
N	0.4569	-1.4246	0.6289
O	-2.3752	-0.9479	0.3267
H	-0.5227	-1.5344	0.8762
C	-2.4992	-0.1945	-0.6368
C	-3.4741	0.9666	-0.6636
H	-2.9338	1.8592	-0.9972
H	-4.2504	0.7326	-1.3992
C	-1.6311	-0.3495	-1.8610
H	-0.7628	0.3037	-1.7654
H	-1.3105	-1.3894	-1.9533
H	-2.1933	-0.0726	-2.7535
C	-4.1054	1.2367	0.6878
H	-3.3477	1.5181	1.4259
H	-4.8300	2.0536	0.6123
H	-4.6271	0.3498	1.0632

Indole-2-pentanone

Conformer 1

C	2.1559	-0.6537	-0.3594
C	1.2339	-0.5263	0.6793
C	1.2886	0.5137	1.6151
C	2.3063	1.4610	1.4717
C	3.2370	1.3635	0.4393
C	3.1770	0.3087	-0.4797
C	1.7939	-1.8121	-1.1007
C	0.6666	-2.3466	-0.5199
H	0.5587	0.5921	2.4144
H	2.3695	2.2872	2.1759
H	4.0187	2.1128	0.3444
H	3.9052	0.2401	-1.2819
H	2.2972	-2.2012	-1.9755
H	0.0628	-3.2047	-0.7814
N	0.3282	-1.5570	0.5490
O	-2.4371	-0.8500	1.1138
H	-0.5069	-1.6642	1.1171
C	-2.9384	0.0849	0.4875
C	-2.0809	1.1475	-0.1649
H	-2.6290	2.0966	-0.1780
H	-1.2051	1.3049	0.4704

C	-4.4300	0.2206	0.3238
H	-4.9258	-0.6847	0.6847
H	-4.7811	1.0777	0.9029
H	-4.6766	0.3558	-0.7321
C	-1.6817	0.7271	-1.5725
H	-2.5633	0.7291	-2.2235
H	-1.3035	-0.3004	-1.5731
C	-0.6238	1.6509	-2.1524
H	-0.3387	1.3184	-3.1550
H	-0.9977	2.6767	-2.2252
H	0.2767	1.6594	-1.5309

Conformer 2

C	2.1389	-0.6725	-0.3664
C	1.2321	-0.5227	0.6827
C	1.3008	0.5369	1.5953
C	2.3165	1.4806	1.4168
C	3.2317	1.3609	0.3728
C	3.1580	0.2869	-0.5225
C	1.7669	-1.8470	-1.0768
C	0.6482	-2.3687	-0.4685
H	0.5831	0.6328	2.4036
H	2.3902	2.3216	2.1022
H	4.0117	2.1081	0.2502
H	3.8742	0.2009	-1.3338
H	2.2581	-2.2554	-1.9497
H	0.0410	-3.2324	-0.7021
N	0.3246	-1.5555	0.5873
O	-2.4455	-0.8530	1.1215
H	-0.5032	-1.6500	1.1682
C	-2.9479	0.0634	0.4650
C	-2.0966	1.1649	-0.1285
H	-2.6695	2.0991	-0.1360
H	-1.2468	1.3289	0.5408
C	-4.4298	0.1363	0.2126
H	-4.9063	-0.7931	0.5343
H	-4.8523	0.9712	0.7763
H	-4.6177	0.2723	-0.8551
C	-1.6366	0.7932	-1.5319
H	-2.4941	0.7851	-2.2140
H	-1.2256	-0.2221	-1.5436
C	-0.5886	1.7649	-2.0506
H	-0.9940	2.7783	-2.1158
H	0.2872	1.7888	-1.3957
H	-0.2555	1.4649	-3.0493

Conformer 3

C	2.1835	-0.6245	-0.3438

C	1.2358	-0.5281	0.6748
C	1.2676	0.4814	1.6442
C	2.2907	1.4297	1.5578
C	3.2474	1.3629	0.5469
C	3.2088	0.3388	-0.4072
C	1.8386	-1.7581	-1.1303
C	0.6968	-2.3089	-0.5945
H	0.5176	0.5355	2.4267
H	2.3379	2.2321	2.2903
H	4.0332	2.1124	0.4968
H	3.9574	0.2939	-1.1921
H	2.3633	-2.1204	-2.0040
H	0.0996	-3.1579	-0.8975
N	0.3331	-1.5529	0.4902
O	-2.4186	-0.8221	1.1042
H	-0.5140	-1.6772	1.0370
C	-2.9222	0.1373	0.5137
C	-2.0709	1.1380	-0.2341
H	-2.5861	2.1042	-0.2648
H	-1.1544	1.2939	0.3429
C	-4.4114	0.3570	0.4911
H	-4.6546	1.2488	1.0736
H	-4.7512	0.4783	-0.5409
H	-4.9197	-0.5064	0.9274
C	-1.7659	0.6380	-1.6402
H	-2.6811	0.6568	-2.2443
H	-1.4375	-0.4059	-1.6135
C	-0.6975	1.4825	-2.3121
H	-1.0195	2.5249	-2.4016
H	0.2368	1.4623	-1.7431
H	-0.4887	1.1014	-3.3167

Conformer 4

C	1.8376	-0.4117	-0.3145
C	1.0206	-0.7773	0.7551
C	0.8603	0.0220	1.8932
C	1.5365	1.2451	1.9245
C	2.3491	1.6430	0.8646
C	2.5146	0.8216	-0.2573
C	1.7582	-1.4547	-1.2783
C	0.8872	-2.4053	-0.7968
H	0.2169	-0.2841	2.7120
H	1.4219	1.8969	2.7877
H	2.8619	2.6007	0.9068
H	3.1481	1.1389	-1.0800
H	2.2621	-1.4899	-2.2348
H	0.5246	-3.3287	-1.2275
N	0.4338	-1.9790	0.4245
O	-2.4918	-0.7803	0.6244

H	-0.2793	-2.4385	0.9766
C	-2.2859	0.4293	0.5403
C	-1.7232	1.0983	-0.7038
H	-2.3135	1.9984	-0.9052
H	-0.7001	1.4132	-0.4767
C	-2.5815	1.3441	1.7021
H	-3.4712	1.9369	1.4778
H	-2.7590	0.7502	2.6027
H	-1.7284	2.0025	1.8839
C	-1.7472	0.1944	-1.9310
H	-2.7854	0.0325	-2.2450
H	-1.3397	-0.7931	-1.6979
C	-0.9553	0.7980	-3.0793
H	-1.3579	1.7765	-3.3629
H	0.0971	0.9278	-2.8071
H	-0.9991	0.1437	-3.9558

Conformer 5

C	1.8820	-0.5424	-0.3481
C	1.0154	-0.7072	0.7318
C	0.8657	0.2549	1.7380
C	1.6155	1.4290	1.6256
C	2.4858	1.6245	0.5547
C	2.6334	0.6453	-0.4359
C	1.7609	-1.7022	-1.1626
C	0.8208	-2.5249	-0.5849
H	0.1779	0.1045	2.5642
H	1.5124	2.2027	2.3829
H	3.0552	2.5479	0.4835
H	3.3093	0.8077	-1.2698
H	2.2881	-1.8997	-2.0864
H	0.4162	-3.4791	-0.8940
N	0.3736	-1.9141	0.5577
O	-2.4105	-1.0055	1.0802
H	-0.3808	-2.2468	1.1474
C	-2.4568	0.0703	0.4792
C	-2.1455	0.1558	-1.0047
H	-1.6247	-0.7677	-1.2852
H	-3.0949	0.1746	-1.5497
C	-2.8836	1.3311	1.1861
H	-2.0198	1.9823	1.3381
H	-3.6478	1.8445	0.5980
H	-3.3105	1.0771	2.1607
C	-1.2944	1.3659	-1.3736
H	-0.4762	1.4951	-0.6606
H	-1.9003	2.2779	-1.3130
C	-0.7170	1.2452	-2.7735
H	-0.0709	0.3658	-2.8596
H	-1.5133	1.1604	-3.5196

H	-0.1169	2.1285	-3.0135

Conformer 6

C	1.8825	-0.5465	-0.3502
C	1.0093	-0.7022	0.7257
C	0.8549	0.2677	1.7238
C	1.6080	1.4394	1.6080
C	2.4862	1.6250	0.5417
C	2.6374	0.6387	-0.4412
C	1.7616	-1.7103	-1.1591
C	0.8157	-2.5267	-0.5818
H	0.1596	0.1258	2.5453
H	1.5004	2.2195	2.3581
H	3.0582	2.5465	0.4676
H	3.3183	0.7941	-1.2724
H	2.2917	-1.9139	-2.0799
H	0.4083	-3.4804	-0.8886
N	0.3651	-1.9084	0.5554
O	-2.4085	-1.0223	1.0806
H	-0.3965	-2.2334	1.1406
C	-2.4570	0.0548	0.4815
C	-2.1237	0.1473	-0.9994
H	-1.5913	-0.7683	-1.2772
H	-3.0663	0.1592	-1.5593
C	-2.9035	1.3117	1.1818
H	-3.3487	1.0523	2.1468
H	-2.0494	1.9673	1.3515
H	-3.6611	1.8221	0.5798
C	-1.2811	1.3683	-1.3564
H	-0.4675	1.4988	-0.6390
H	-1.8931	2.2755	-1.2923
C	-0.6960	1.2606	-2.7549
H	-0.0421	0.3875	-2.8433
H	-1.4886	1.1731	-3.5042
H	-0.1023	2.1506	-2.9867

Conformer 7

C	2.1569	-0.6680	-0.3499
C	1.2320	-0.4955	0.6795
C	1.2884	0.5795	1.5746
C	2.3153	1.5119	1.4017
C	3.2521	1.3669	0.3805
C	3.1875	0.2799	-0.4997
C	1.7848	-1.8465	-1.0538
C	0.6504	-2.3495	-0.4593
H	0.5518	0.6964	2.3629
H	2.3802	2.3645	2.0737
H	4.0417	2.1049	0.2634

H	3.9193	0.1756	-1.2947
H	2.2854	-2.2681	-1.9152
H	0.0372	-3.2085	-0.6951
N	0.3215	-1.5252	0.5864
O	-2.4679	-0.8738	1.1259
H	-0.5130	-1.6080	1.1600
C	-3.0087	-0.1458	0.2890
C	-2.3293	0.1842	-1.0233
H	-1.6920	-0.6636	-1.2939
H	-3.0872	0.2668	-1.8102
C	-4.3620	0.4665	0.5288
H	-5.0938	0.0013	-0.1354
H	-4.6655	0.3016	1.5662
H	-4.3222	1.5430	0.3457
C	-1.5264	1.4709	-0.8978
H	-0.9443	1.4688	0.0303
H	-2.2056	2.3275	-0.8272
C	-0.5914	1.6643	-2.0792
H	-1.1552	1.7451	-3.0134
H	-0.0045	2.5793	-1.9528
H	0.1064	0.8268	-2.1745

Conformer 8

C	2.1338	-0.6869	-0.3685
C	1.2244	-0.4665	0.6647
C	1.3028	0.6415	1.5181
C	2.3371	1.5558	1.2976
C	3.2602	1.3615	0.2728
C	3.1728	0.2440	-0.5662
C	1.7384	-1.8839	-1.0263
C	0.6071	-2.3523	-0.4005
H	0.5738	0.7999	2.3056
H	2.4172	2.4337	1.9347
H	4.0567	2.0854	0.1192
H	3.8914	0.1033	-1.3671
H	2.2222	-2.3405	-1.8801
H	-0.0211	-3.2104	-0.5994
N	0.2988	-1.4867	0.6189
O	-2.5156	-0.8851	1.1329
H	-0.5349	-1.5346	1.1959
C	-3.0257	-0.1959	0.2488
C	-2.2474	0.2016	-0.9867
H	-1.5609	-0.6104	-1.2334
H	-2.9388	0.2890	-1.8344
C	-4.4400	0.3086	0.3446
H	-4.4645	1.3837	0.1422
H	-5.0619	-0.2188	-0.3837
H	-4.8331	0.1361	1.3495
C	-1.5143	1.5117	-0.7455

H	-0.9763	1.4766	0.2072
H	-2.2332	2.3322	-0.6518
C	-0.5383	1.8175	-1.8689
H	-1.0612	1.8977	-2.8261
H	-0.0222	2.7623	-1.6770
H	0.2175	1.0319	-1.9608

Conformer 9

C	1.8614	-0.3351	-0.3078
C	1.0203	-0.7366	0.7297
C	0.8816	-0.0088	1.9175
C	1.6107	1.1775	2.0375
C	2.4518	1.6089	1.0137
C	2.5910	0.8602	-0.1615
C	1.7406	-1.3027	-1.3430
C	0.8245	-2.2440	-0.9328
H	0.2133	-0.3387	2.7067
H	1.5150	1.7737	2.9421
H	3.0076	2.5364	1.1264
H	3.2453	1.2052	-0.9562
H	2.2467	-1.2921	-2.2990
H	0.4210	-3.1157	-1.4301
N	0.3825	-1.8850	0.3140
O	-2.4602	-0.7211	0.7611
H	-0.3617	-2.3418	0.8265
C	-2.2209	0.4667	0.5577
C	-1.7211	0.9982	-0.7756
H	-2.2364	1.9433	-0.9851
H	-0.6551	1.2223	-0.6670
C	-2.3917	1.4901	1.6514
H	-1.4941	2.1084	1.7266
H	-3.2586	2.1161	1.4266
H	-2.5483	0.9853	2.6079
C	-1.9564	0.0316	-1.9286
H	-3.0304	-0.0356	-2.1344
H	-1.6304	-0.9787	-1.6688
C	-1.2279	0.4803	-3.1851
H	-1.4274	-0.2113	-4.0094
H	-1.5534	1.4810	-3.4911
H	-0.1446	0.5109	-3.0225

Conformer 10

C	2.0655	-0.5489	-0.2419
C	1.0831	0.2887	-0.7688
C	0.9042	1.6098	-0.3387
C	1.7431	2.0791	0.6757
C	2.7321	1.2637	1.2222
C	2.9079	-0.0500	0.7694

C	1.9461	-1.8023	-0.9027
C	0.8979	-1.7108	-1.7901
H	0.1262	2.2388	-0.7585
H	1.6198	3.0943	1.0444
H	3.3766	1.6491	2.0084
H	3.6794	-0.6794	1.2017
H	2.5485	-2.6839	-0.7281
H	0.4587	-2.4389	-2.4587
N	0.3744	-0.4472	-1.6938
O	-2.4340	0.3649	-1.2378
H	-0.4688	-0.1239	-2.1562
C	-2.5698	0.7428	-0.0699
C	-1.9645	-0.0025	1.1103
H	-0.9840	0.4393	1.3090
H	-2.5836	0.1778	1.9943
C	-3.2955	2.0159	0.2586
H	-2.6714	2.6384	0.9038
H	-3.5085	2.5698	-0.6608
H	-4.2373	1.7773	0.7615
C	-1.8518	-1.5061	0.8775
H	-1.4841	-1.7274	-0.1261
H	-2.8489	-1.9573	0.9432
C	-0.9277	-2.1479	1.8985
H	-1.2880	-1.9767	2.9173
H	-0.8680	-3.2270	1.7304
H	0.0845	-1.7391	1.8237

Indole-cyclohexanone

Conformer 1

C	2.2417	-0.1205	0.4330
C	1.4539	-0.3415	-0.6962
C	1.0625	0.6873	-1.5602
C	1.4790	1.9850	-1.2494
C	2.2572	2.2385	-0.1217
C	2.6506	1.1949	0.7250
C	2.4319	-1.3814	1.0632
C	1.7499	-2.3250	0.3290
H	0.4449	0.4893	-2.4307
H	1.1831	2.8089	-1.8947
H	2.5632	3.2567	0.1052
H	3.2558	1.4008	1.6025
H	2.9934	-1.5719	1.9680
H	1.6209	-3.3891	0.4727
N	1.1579	-1.6866	-0.7295
O	-1.7574	-1.6145	-1.3159
H	0.5307	-2.1147	-1.4015
C	-2.1335	-0.8201	-0.4509
C	-2.8854	0.4438	-0.8082
C	-2.0091	-1.1326	1.0232

C	-2.3725	1.6282	-0.0004
H	-3.9492	0.2715	-0.6091
H	-2.7656	0.6433	-1.8778
C	-1.5039	0.0806	1.7912
H	-2.9954	-1.4398	1.3874
H	-1.3218	-1.9741	1.1572
C	-2.3292	1.3302	1.4962
H	-3.0072	2.5024	-0.1846
H	-1.3659	1.8934	-0.3402
H	-1.5206	-0.1304	2.8655
H	-0.4606	0.2771	1.5244
H	-1.8954	2.1864	2.0258
H	-3.3487	1.1978	1.8769

Conformer 2

C	2.1707	-0.2853	-0.7281
C	1.6803	-0.3326	0.5763
C	1.4632	0.8174	1.3458
C	1.7469	2.0518	0.7551
C	2.2358	2.1290	-0.5470
C	2.4563	0.9684	-1.2991
C	2.2417	-1.6268	-1.1957
C	1.7773	-2.4479	-0.1950
H	1.0570	0.7553	2.3507
H	1.5755	2.9649	1.3208
H	2.4506	3.1002	-0.9863
H	2.8324	1.0366	-2.3158
H	2.5728	-1.9518	-2.1738
H	1.6401	-3.5159	-0.1504
N	1.4325	-1.6531	0.8720
O	-1.4012	-1.2425	1.6416
H	0.9750	-1.9733	1.7170
C	-1.6660	-0.4425	0.7378
C	-2.0483	0.9924	1.0317
C	-1.5276	-0.7963	-0.7249
C	-3.2995	1.3593	0.2488
H	-1.2158	1.6396	0.7348
H	-2.2099	1.1185	2.1068
C	-2.7869	-0.3970	-1.4758
H	-0.6700	-0.2507	-1.1314
H	-1.3345	-1.8670	-0.8296
C	-3.1161	1.0780	-1.2432
H	-3.5354	2.4171	0.4024
H	-4.1488	0.7753	0.6239
H	-2.6537	-0.5852	-2.5460
H	-3.6259	-1.0146	-1.1347
H	-4.0290	1.3435	-1.7863
H	-2.3079	1.7051	-1.6409

Conformer 3

C	2.6725	-0.3452	-0.5276
C	1.7044	-0.2719	0.4723
C	1.2936	0.9400	1.0417
C	1.8804	2.1130	0.5638
C	2.8480	2.0734	-0.4345
C	3.2600	0.8500	-0.9861
C	2.8277	-1.7193	-0.8593
C	1.9507	-2.4414	-0.0814
H	0.5387	0.9694	1.8203
H	1.5816	3.0712	0.9810
H	3.3031	2.9934	-0.7891
H	4.0204	0.8285	-1.7614
H	3.5012	-2.1322	-1.5984
H	1.7409	-3.5013	-0.0326
N	1.2757	-1.5585	0.7200
O	-1.4811	-1.2002	1.4838
H	0.5162	-1.7931	1.3531
C	-1.9259	-0.3738	0.6855
C	-2.7240	0.8281	1.1379
C	-1.5872	-0.4178	-0.7892
C	-3.9656	0.9766	0.2690
H	-2.0813	1.7112	1.0525
H	-3.0033	0.7081	2.1888
C	-2.8444	-0.2439	-1.6202
H	-0.8730	0.3866	-0.9979
H	-1.1052	-1.3734	-1.0242
C	-3.6003	1.0204	-1.2155
H	-4.5057	1.8883	0.5462
H	-4.6416	0.1309	0.4477
H	-2.5804	-0.1973	-2.6821
H	-3.4965	-1.1157	-1.4847
H	-4.5084	1.1171	-1.8191
H	-2.9799	1.9021	-1.4165

Conformer 4

C	2.6725	-0.3452	-0.5276
C	1.7044	-0.2719	0.4723
C	1.2936	0.9400	1.0417
C	1.8804	2.1130	0.5638
C	2.8480	2.0734	-0.4345
C	3.2600	0.8500	-0.9861
C	2.8277	-1.7193	-0.8593
C	1.9507	-2.4414	-0.0814
H	0.5387	0.9694	1.8203
H	1.5816	3.0712	0.9810
H	3.3031	2.9934	-0.7891
H	4.0204	0.8285	-1.7614

H	3.5012	-2.1322	-1.5984
H	1.7409	-3.5013	-0.0326
N	1.2757	-1.5585	0.7200
O	-1.4811	-1.2002	1.4838
H	0.5162	-1.7931	1.3531
C	-1.9259	-0.3738	0.6855
C	-2.7240	0.8281	1.1379
C	-1.5872	-0.4178	-0.7892
C	-3.9656	0.9766	0.2690
H	-2.0813	1.7112	1.0525
H	-3.0033	0.7081	2.1888
C	-2.8444	-0.2439	-1.6202
H	-0.8730	0.3866	-0.9979
H	-1.1052	-1.3734	-1.0242
C	-3.6003	1.0204	-1.2155
H	-4.5057	1.8883	0.5462
H	-4.6416	0.1309	0.4477
H	-2.5804	-0.1973	-2.6821
H	-3.4965	-1.1157	-1.4847
H	-4.5084	1.1171	-1.8191
H	-2.9799	1.9021	-1.4165

Conformer 4

C	2.7777	-0.3633	-0.4860
C	1.7409	-0.2542	0.4391
C	1.3163	0.9747	0.9593
C	1.9623	2.1277	0.5068
C	3.0020	2.0495	-0.4168
C	3.4232	0.8116	-0.9182
C	2.9286	-1.7457	-0.7843
C	1.9830	-2.4374	-0.0607
H	0.5111	1.0323	1.6844
H	1.6506	3.0981	0.8854
H	3.4966	2.9582	-0.7509
H	4.2364	0.7592	-1.6350
H	3.6455	-2.1839	-1.4651
H	1.7496	-3.4922	-0.0107
N	1.2720	-1.5285	0.6781
O	-1.4691	-1.1720	1.4424
H	0.4721	-1.7394	1.2683
C	-1.9855	-0.3575	0.6772
C	-2.8880	0.7482	1.1772
C	-1.6275	-0.3093	-0.7933
C	-4.1222	0.8547	0.2917
H	-2.3173	1.6830	1.1675
H	-3.1818	0.5380	2.2108
C	-2.8859	-0.1787	-1.6398
H	-0.9668	0.5483	-0.9560
H	-1.0864	-1.2191	-1.0668

C	-3.7419	0.9987	-1.1822
H	-4.7290	1.7113	0.6037
H	-4.7395	-0.0431	0.4158
H	-2.6088	-0.0518	-2.6919
H	-3.4708	-1.1029	-1.5665
H	-4.6489	1.0532	-1.7939
H	-3.1933	1.9373	-1.3324

Conformer 5

C	2.8546	-0.1618	0.1737
C	1.6983	-0.3325	-0.5852
C	1.1440	0.6917	-1.3627
C	1.7844	1.9337	-1.3498
C	2.9426	2.1338	-0.6012
C	3.4911	1.0947	0.1604
C	3.1161	-1.3996	0.8233
C	2.1189	-2.2793	0.4678
H	0.2464	0.5311	-1.9501
H	1.3740	2.7526	-1.9357
H	3.4291	3.1057	-0.6096
H	4.3948	1.2588	0.7388
H	3.9404	-1.6172	1.4893
H	1.9359	-3.3072	0.7495
N	1.2687	-1.6272	-0.3867
O	-1.5622	-1.5504	-0.8324
H	0.4092	-2.0074	-0.7740
C	-2.0385	-0.5688	-0.2643
C	-1.5124	-0.0865	1.0705
C	-3.0710	0.3147	-0.9306
C	-2.6631	0.2488	2.0057
H	-0.8910	0.7974	0.8927
H	-0.8853	-0.8651	1.5149
C	-4.1919	0.6426	0.0460
H	-2.5694	1.2274	-1.2703
H	-3.4755	-0.1998	-1.8080
C	-3.6488	1.2161	1.3538
H	-2.2695	0.6855	2.9301
H	-3.1896	-0.6732	2.2816
H	-4.8847	1.3564	-0.4132
H	-4.7634	-0.2683	0.2640
H	-4.4781	1.4137	2.0417
H	-3.1529	2.1751	1.1599

Conformer 6

C	3.0429	-0.1099	0.0932
C	1.8098	-0.2275	-0.5468
C	1.2803	0.7789	-1.3616
C	2.0313	1.9465	-1.5188
C	3.2675	2.0933	-0.8926

C	3.7863	1.0718	-0.0861
C	3.2482	-1.3108	0.8282
C	2.1509	-2.1181	0.6301
H	0.3207	0.6603	-1.8537
H	1.6461	2.7502	-2.1422
H	3.8386	3.0074	-1.0326
H	4.7507	1.1940	0.3977
H	4.1057	-1.5509	1.4420
H	1.9056	-3.0996	1.0111
N	1.2906	-1.4608	-0.2099
O	-1.5413	-1.4896	-0.7111
H	0.3791	-1.7980	-0.5096
C	-2.1984	-0.5596	-0.2432
C	-1.6396	0.3530	0.8238
C	-3.5597	-0.1803	-0.7864
C	-2.6523	0.5243	1.9449
H	-1.4009	1.3145	0.3574
H	-0.7164	-0.0736	1.2254
C	-4.5405	0.0065	0.3644
H	-3.4490	0.7465	-1.3595
H	-3.9147	-0.9619	-1.4654
C	-4.0031	0.9893	1.4056
H	-2.2732	1.2456	2.6774
H	-2.7820	-0.4312	2.4687
H	-5.5017	0.3644	-0.0207
H	-4.7259	-0.9612	0.8475
H	-4.7170	1.0813	2.2315
H	-3.8979	1.9838	0.9552

Conformer 7

C	3.0839	-0.0880	0.0740
C	1.8515	-0.1930	-0.5688
C	1.3499	0.8065	-1.4119
C	2.1348	1.9470	-1.6017
C	3.3724	2.0780	-0.9752
C	3.8607	1.0676	-0.1372
C	3.2553	-1.2717	0.8447
C	2.1375	-2.0547	0.6657
H	0.3887	0.6984	-1.9039
H	1.7738	2.7418	-2.2491
H	3.9694	2.9717	-1.1399
H	4.8265	1.1768	0.3463
H	4.1040	-1.5168	1.4685
H	1.8643	-3.0178	1.0747
N	1.2989	-1.4007	-0.1985
O	-1.5217	-1.4405	-0.7108
H	0.3829	-1.7250	-0.4982
C	-2.2529	-0.5692	-0.2395
C	-1.7180	0.4995	0.6887

C	-3.6991	-0.4179	-0.6612
C	-2.6386	0.6691	1.8854
H	-1.6300	1.4316	0.1190
H	-0.7198	0.2143	1.0340
C	-4.5863	-0.2201	0.5605
H	-3.7673	0.4408	-1.3367
H	-4.0105	-1.3120	-1.2109
C	-4.0811	0.9139	1.4525
H	-2.2881	1.5033	2.5030
H	-2.5971	-0.2335	2.5072
H	-5.6134	-0.0092	0.2413
H	-4.6155	-1.1486	1.1446
H	-4.7202	0.9972	2.3395
H	-4.1501	1.8669	0.9140

Conformer 8

C	2.9574	-0.3069	-0.5836
C	1.9272	-0.1495	0.3418
C	1.7739	1.0090	1.1133
C	2.7032	2.0375	0.9283
C	3.7450	1.9063	0.0131
C	3.8861	0.7393	-0.7485
C	2.7952	-1.5888	-1.1770
C	1.6801	-2.1719	-0.6183
H	0.9622	1.1091	1.8269
H	2.6104	2.9514	1.5094
H	4.4594	2.7162	-0.1125
H	4.7006	0.6442	-1.4588
H	3.4253	-2.0317	-1.9365
H	1.2083	-3.1290	-0.7930
N	1.1647	-1.2983	0.3038
O	-1.5343	-1.0966	1.3689
H	0.3224	-1.4484	0.8536
C	-2.3159	-0.3250	0.8193
C	-3.8062	-0.3367	1.0939
C	-1.8420	0.7638	-0.1152
C	-4.5641	-0.3789	-0.2293
H	-4.0558	0.5690	1.6586
H	-4.0625	-1.2042	1.7113
C	-2.6194	0.6911	-1.4186
H	-1.9850	1.7289	0.3843
H	-0.7772	0.6407	-0.3147
C	-4.1241	0.7483	-1.1660
H	-5.6414	-0.3063	-0.0437
H	-4.3844	-1.3422	-0.7204
H	-2.3167	1.5135	-2.0757
H	-2.3754	-0.2449	-1.9380
H	-4.6607	0.6711	-2.1173
H	-4.3852	1.7182	-0.7238

Conformer 9

C	2.7982	-0.1047	-0.7111
C	1.8833	-0.2369	0.3341
C	1.8652	0.6239	1.4371
C	2.8144	1.6496	1.4644
C	3.7399	1.8033	0.4398
C	3.7450	0.9315	-0.6560
C	2.5172	-1.1350	-1.6461
C	1.4483	-1.8514	-1.1623
H	1.1411	0.5031	2.2363
H	2.8253	2.3375	2.3068
H	4.4714	2.6052	0.4850
H	4.4722	1.0571	-1.4534
H	3.0406	-1.3235	-2.5736
H	0.9200	-2.7084	-1.5623
N	1.0684	-1.3044	0.0321
O	-1.5888	-1.4168	1.2104
H	0.2780	-1.6035	0.5957
C	-2.3388	-0.5262	0.8131
C	-3.8436	-0.6626	0.8033
C	-1.8220	0.8094	0.3335
C	-4.3501	-0.3831	-0.6066
H	-4.2550	0.0653	1.5120
H	-4.1305	-1.6649	1.1337
C	-2.3416	1.0609	-1.0727
H	-2.1705	1.5802	1.0278
H	-0.7339	0.8157	0.3449
C	-3.8684	0.9821	-1.1055
H	-5.4440	-0.4206	-0.6223
H	-3.9842	-1.1614	-1.2885
H	-2.0076	2.0448	-1.4182
H	-1.9264	0.3128	-1.7603
H	-4.2275	1.1548	-2.1255
H	-4.2898	1.7739	-0.4736

Conformer 10

C	2.9502	0.0641	0.1544
C	1.8305	-0.0887	-0.6622
C	1.5053	0.8193	-1.6784
C	2.3507	1.9177	-1.8588
C	3.4781	2.0939	-1.0599
C	3.7913	1.1749	-0.0510
C	2.9593	-1.0310	1.0640
C	1.8528	-1.8052	0.7944
H	0.6263	0.6799	-2.2989
H	2.1244	2.6432	-2.6369
H	4.1255	2.9535	-1.2203
H	4.6725	1.3190	0.5663

H	3.6893	-1.2208	1.8351
H	1.4863	-2.7126	1.2540
N	1.1794	-1.2339	-0.2522
O	-1.6485	-1.4755	-0.9060
H	0.3044	-1.5689	-0.6456
C	-2.3949	-0.6611	-0.3623
C	-1.8902	0.6697	0.1434
C	-3.8822	-0.8861	-0.2077
C	-2.2874	0.8291	1.6027
H	-2.3234	1.4583	-0.4800
H	-0.8070	0.7197	0.0435
C	-4.2650	-0.6912	1.2553
H	-4.4017	-0.1644	-0.8470
H	-4.1439	-1.8931	-0.5462
C	-3.7953	0.6691	1.7777
H	-1.9669	1.8123	1.9656
H	-1.7686	0.0751	2.2087
H	-5.3500	-0.7788	1.3716
H	-3.8079	-1.4836	1.8604
H	-4.0567	0.7697	2.8369
H	-4.3141	1.4698	1.2372

Indole-acetophenone

Conformer 1

C	2.0558	0.0322	-0.3855
C	1.2099	1.0854	-0.7324
C	0.9064	2.1329	0.1453
C	1.4714	2.0897	1.4233
C	2.3105	1.0435	1.8023
C	2.6154	0.0115	0.9064
C	2.1345	-0.8308	-1.5134
C	1.3295	-0.3077	-2.4996
H	0.2453	2.9416	-0.1486
H	1.2498	2.8841	2.1320
H	2.7353	1.0261	2.8026
H	3.2671	-0.8023	1.2086
H	2.7026	-1.7484	-1.5842
H	1.0907	-0.6627	-3.4926
N	0.7612	0.8405	-2.0119
O	-2.4625	1.0155	-1.3708
H	0.0698	1.4020	-2.4924
C	-2.3927	0.7448	-0.1705
C	-2.9150	1.6973	0.8734
H	-3.5081	1.1593	1.6190
H	-2.0859	2.2281	1.3482
H	-3.5708	2.4307	0.3938
C	-1.7550	-0.5269	0.2747
C	-1.2705	-0.6922	1.5777

C	-1.5836	-1.5537	-0.6617
C	-0.6073	-1.8692	1.9320
H	-1.3543	0.0965	2.3185
C	-0.9224	-2.7293	-0.3022
H	-1.9366	-1.4296	-1.6830
C	-0.4305	-2.8839	0.9922
H	-0.2020	-1.9824	2.9343
H	-0.7672	-3.5102	-1.0418
H	0.1082	-3.7882	1.2636

Conformer 2

C	1.9643	0.0130	-0.6553
C	1.3200	1.2148	-0.3603
C	1.4911	1.8846	0.8572
C	2.3378	1.2998	1.8042
C	2.9889	0.0966	1.5398
C	2.8145	-0.5552	0.3130
C	1.5536	-0.3733	-1.9615
C	0.6714	0.5768	-2.4220
H	0.9716	2.8127	1.0711
H	2.4806	1.7877	2.7654
H	3.6340	-0.3455	2.2945
H	3.3189	-1.4960	0.1173
H	1.8620	-1.2608	-2.4972
H	0.1185	0.6556	-3.3480
N	0.5272	1.5260	-1.4427
O	-2.7803	1.1883	-1.1399
H	-0.1071	2.3135	-1.4834
C	-2.5473	-0.0160	-1.0685
C	-2.9237	-0.9549	-2.1864
H	-3.6231	-0.4491	-2.8595
H	-2.0349	-1.2384	-2.7568
H	-3.4272	-1.8407	-1.7901
C	-1.8495	-0.5709	0.1280
C	-1.2158	-1.8197	0.1034
C	-1.7777	0.2184	1.2828
C	-0.5045	-2.2636	1.2206
H	-1.2207	-2.4421	-0.7860
C	-1.0697	-0.2314	2.3977
H	-2.2487	1.1982	1.3072
C	-0.4292	-1.4688	2.3636
H	0.0194	-3.2155	1.1889
H	-0.9893	0.3990	3.2789
H	0.1528	-1.8010	3.2191

Conformer 3

C	1.9868	0.0536	-0.6758
C	1.3098	1.2206	-0.3229

C	1.4492	1.8243	0.9325
C	2.2989	1.2072	1.8566
C	2.9832	0.0372	1.5334
C	2.8402	-0.5478	0.2698
C	1.6000	-0.2692	-2.0068
C	0.7013	0.6835	-2.4245
H	0.9034	2.7251	1.1925
H	2.4165	1.6424	2.8465
H	3.6303	-0.4307	2.2705
H	3.3708	-1.4627	0.0277
H	1.9357	-1.1177	-2.5867
H	0.1561	0.8008	-3.3515
N	0.5233	1.5755	-1.3961
O	-2.7905	1.1009	-1.2368
H	-0.1273	2.3491	-1.4036
C	-2.4864	-0.0870	-1.1330
C	-2.7403	-1.0611	-2.2545
H	-1.8058	-1.2983	-2.7674
H	-3.2133	-1.9700	-1.8725
H	-3.4283	-0.6066	-2.9741
C	-1.8268	-0.5822	0.1094
C	-1.1147	-1.7867	0.1386
C	-1.8778	0.2198	1.2569
C	-0.4473	-2.1757	1.3035
H	-1.0224	-2.4140	-0.7426
C	-1.2147	-0.1759	2.4205
H	-2.4113	1.1673	1.2414
C	-0.4973	-1.3700	2.4402
H	0.1376	-3.0917	1.3141
H	-1.2338	0.4624	3.2990
H	0.0485	-1.6602	3.3346

Conformer 4

C	1.9016	0.1178	-0.8206
C	1.2904	1.1954	-0.1787
C	1.5364	1.5139	1.1624
C	2.4252	0.6976	1.8692
C	3.0472	-0.3875	1.2551
C	2.7983	-0.6858	-0.0901
C	1.4015	0.0839	-2.1521
C	0.5020	1.1159	-2.2881
H	1.0313	2.3409	1.6501
H	2.6173	0.9032	2.9196
H	3.7221	-1.0179	1.8280
H	3.2745	-1.5419	-0.5572
H	1.6558	-0.6360	-2.9180
H	-0.1193	1.4244	-3.1181
N	0.4345	1.7765	-1.0881
O	-2.8165	1.3319	-0.9037

H	-0.2047	2.5321	-0.8764
C	-2.5161	0.1416	-0.9955
C	-2.8209	-0.6540	-2.2320
H	-1.9101	-0.8234	-2.8127
H	-3.2905	-1.6061	-1.9685
H	-3.5289	-0.0955	-2.8531
C	-1.8022	-0.5313	0.1296
C	-1.8081	0.0836	1.3905
C	-1.0740	-1.7133	-0.0595
C	-1.0914	-0.4775	2.4471
H	-2.3427	1.0185	1.5384
C	-0.3515	-2.2656	0.9988
H	-1.0025	-2.1843	-1.0366
C	-0.3628	-1.6488	2.2499
H	-1.0716	0.0214	3.4112
H	0.2543	-3.1532	0.8383
H	0.2299	-2.0609	3.0617

Conformer 5

C	1.9634	0.2335	-0.9259
C	1.2906	1.2265	-0.2125
C	1.4626	1.4158	1.1633
C	2.3423	0.5574	1.8299
C	3.0241	-0.4461	1.1446
C	2.8467	-0.6171	-0.2332
C	1.5413	0.3315	-2.2803
C	0.6268	1.3564	-2.3614
H	0.9183	2.1843	1.7023
H	2.4856	0.6689	2.9018
H	3.6964	-1.1073	1.6851
H	3.3775	-1.4042	-0.7594
H	1.8653	-0.2909	-3.1033
H	0.0563	1.7462	-3.1936
N	0.4750	1.8855	-1.1058
O	-2.7955	1.2443	-0.9382
H	-0.1762	2.6190	-0.8580
C	-2.4083	0.0794	-1.0142
C	-2.5209	-0.7049	-2.2959
H	-2.9547	-1.6900	-2.1042
H	-3.1905	-0.1758	-2.9807
H	-1.5423	-0.8012	-2.7707
C	-1.7956	-0.5781	0.1761
C	-1.9926	0.0041	1.4359
C	-0.9952	-1.7190	0.0589
C	-1.3910	-0.5512	2.5669
H	-2.6006	0.8996	1.5371
C	-0.3907	-2.2671	1.1921
H	-0.7878	-2.1706	-0.9068
C	-0.5883	-1.6837	2.4435
H	-1.5317	-0.0862	3.5392

H	0.2614	-3.1318	1.0949
H	-0.0967	-2.0993	3.3191

Conformer 6

C	2.0337	0.2608	-0.7825
C	1.1728	1.2627	-0.3320
C	1.0444	1.5948	1.0216
C	1.8134	0.8745	1.9409
C	2.6785	-0.1332	1.5205
C	2.8011	-0.4484	0.1619
C	1.8884	0.1893	-2.1950
C	0.9477	1.1226	-2.5685
H	0.3467	2.3562	1.3545
H	1.7127	1.0893	3.0022
H	3.2523	-0.6920	2.2553
H	3.4674	-1.2441	-0.1565
H	2.4043	-0.4872	-2.8630
H	0.5287	1.3726	-3.5333
N	0.5118	1.7573	-1.4341
O	-2.5701	0.8923	-1.4192
H	-0.2347	2.4395	-1.3984
C	-2.0923	-0.2273	-1.2578
C	-1.8748	-1.1783	-2.4078
H	-2.2785	-2.1633	-2.1693
H	-2.4098	-0.7989	-3.2829
H	-0.8114	-1.2527	-2.6541
C	-1.6862	-0.6709	0.1131
C	-0.7889	-1.7234	0.3155
C	-2.1872	0.0320	1.2169
C	-0.3960	-2.0680	1.6102
H	-0.3375	-2.2527	-0.5187
C	-1.7943	-0.3179	2.5095
H	-2.8666	0.8691	1.0726
C	-0.8991	-1.3673	2.7063
H	0.3371	-2.8589	1.7627
H	-2.1696	0.2446	3.3634
H	-0.5701	-1.6227	3.7112

Conformer 7

C	2.0327	0.2799	-0.8005
C	1.1567	1.2727	-0.3623
C	0.9864	1.5852	0.9913
C	1.7346	0.8583	1.9228
C	2.6166	-0.1398	1.5144
C	2.7766	-0.4386	0.1558
C	1.9268	0.2246	-2.2175
C	0.9932	1.1581	-2.6055
H	0.2724	2.3350	1.3148

H	1.6002	1.0560	2.9837
H	3.1714	-0.7061	2.2581
H	3.4521	-1.2298	-0.1531
H	2.4595	-0.4459	-2.8784
H	0.5966	1.4156	-3.5780
N	0.5235	1.7804	-1.4761
O	-2.5055	0.8945	-1.4097
H	-0.2342	2.4503	-1.4512
C	-2.0278	-0.2241	-1.2437
C	-1.7812	-1.1555	-2.4025
H	-2.2942	-0.7684	-3.2888
H	-0.7134	-1.2267	-2.6202
H	-2.1908	-2.1442	-2.1864
C	-1.6619	-0.6807	0.1280
C	-0.7608	-1.7306	0.3395
C	-2.1884	0.0092	1.2278
C	-0.3955	-2.0877	1.6399
H	-0.2812	-2.2410	-0.4910
C	-1.8231	-0.3544	2.5256
H	-2.8630	0.8486	1.0750
C	-0.9281	-1.4031	2.7298
H	0.3402	-2.8715	1.7983
H	-2.2174	0.1989	3.3728
H	-0.6202	-1.6647	3.7389

Conformer 8

C	1.7720	1.3755	-0.0227
C	1.4005	0.4793	-1.0249
C	1.6513	-0.8950	-0.9407
C	2.2891	-1.3664	0.2104
C	2.6643	-0.4960	1.2318
C	2.4157	0.8777	1.1266
C	1.3586	2.6692	-0.4447
C	0.7354	2.5336	-1.6643
H	1.3346	-1.5781	-1.7219
H	2.4725	-2.4328	0.3180
H	3.1431	-0.8886	2.1250
H	2.7043	1.5482	1.9299
H	1.4885	3.5965	0.0968
H	0.2615	3.2583	-2.3125
N	0.7543	1.2050	-2.0008
O	-2.3146	0.2166	-1.8689
H	0.3091	0.8010	-2.8152
C	-2.2106	0.4821	-0.6698
C	-2.5511	1.8464	-0.1364
H	-3.0665	2.4195	-0.9169
H	-3.2300	1.7632	0.7168
H	-1.6464	2.3867	0.1467
C	-1.7227	-0.5576	0.2821

C	-1.2298	-0.2255	1.5494
C	-1.7158	-1.8964	-0.1319
C	-0.7331	-1.2244	2.3905
H	-1.1761	0.8061	1.8843
C	-1.2205	-2.8920	0.7124
H	-2.0782	-2.1651	-1.1219
C	-0.7284	-2.5548	1.9723
H	-0.3188	-0.9588	3.3598
H	-1.2015	-3.9263	0.3787
H	-0.3209	-3.3258	2.6211

Conformer 9

C	1.8125	1.3739	-0.2530
C	1.4322	0.3340	-1.1014
C	1.6783	-1.0116	-0.8047
C	2.3217	-1.3003	0.4019
C	2.7054	-0.2829	1.2732
C	2.4596	1.0586	0.9569
C	1.4086	2.5881	-0.8739
C	0.7865	2.2671	-2.0585
H	1.3628	-1.8064	-1.4728
H	2.5109	-2.3371	0.6695
H	3.1947	-0.5326	2.2109
H	2.7597	1.8453	1.6419
H	1.5500	3.5885	-0.4865
H	0.3247	2.8858	-2.8170
N	0.7928	0.9023	-2.1809
O	-2.2954	-0.0952	-1.8721
H	0.3544	0.3799	-2.9283
C	-2.2149	0.3502	-0.7278
C	-2.5716	1.7820	-0.4171
H	-1.6704	2.3617	-0.2049
H	-3.0714	2.2231	-1.2843
H	-3.2641	1.8271	0.4272
C	-1.7500	-0.5277	0.3840
C	-1.7483	-1.9147	0.1864
C	-1.2825	-0.0032	1.5950
C	-1.2840	-2.7681	1.1889
H	-2.0983	-2.3339	-0.7547
C	-0.8159	-0.8606	2.5947
H	-1.2341	1.0676	1.7684
C	-0.8165	-2.2402	2.3914
H	-1.2752	-3.8425	1.0255
H	-0.4291	-0.4494	3.5237
H	-0.4382	-2.9029	3.1655

Conformer 10

C	1.8138	1.3752	-0.2657

C	1.4302	0.3409	-1.1196
C	1.6587	-1.0075	-0.8231
C	2.2875	-1.3056	0.3892
C	2.6744	-0.2940	1.2659
C	2.4469	1.0507	0.9496
C	1.4288	2.5948	-0.8876
C	0.8118	2.2826	-2.0776
H	1.3397	-1.7976	-1.4949
H	2.4613	-2.3451	0.6571
H	3.1515	-0.5510	2.2079
H	2.7494	1.8328	1.6389
H	1.5780	3.5928	-0.4978
H	0.3628	2.9071	-2.8381
N	0.8059	0.9174	-2.2030
O	-2.2761	-0.0847	-1.8675
H	0.3677	0.4000	-2.9544
C	-2.1863	0.3598	-0.7220
C	-2.5176	1.7972	-0.4121
H	-3.2147	1.8533	0.4295
H	-1.6088	2.3598	-0.1956
H	-3.0067	2.2482	-1.2813
C	-1.7368	-0.5268	0.3883
C	-1.7481	-1.9135	0.1856
C	-1.2693	-0.0115	1.6031
C	-1.2975	-2.7752	1.1872
H	-2.0976	-2.3258	-0.7587
C	-0.8170	-0.8773	2.6021
H	-1.2104	1.0579	1.7802
C	-0.8310	-2.2560	2.3938
H	-1.2988	-3.8489	1.0197
H	-0.4309	-0.4735	3.5344
H	-0.4636	-2.9257	3.1676

Indole-benzophenone

Conformer 1

C	3.0899	0.7118	0.0143
C	1.9163	1.1890	0.5999
C	0.9990	1.9935	-0.0871
C	1.2898	2.3147	-1.4161
C	2.4559	1.8546	-2.0256
C	3.3644	1.0541	-1.3229
C	3.7467	-0.0914	0.9872
C	2.9630	-0.1097	2.1176
H	0.0706	2.3120	0.3774
H	0.5865	2.9174	-1.9851
H	2.6611	2.1128	-3.0613
H	4.2615	0.6836	-1.8092
H	4.6735	-0.6334	0.8523
H	3.0719	-0.6340	3.0576

N	1.8502	0.6520	1.8687
O	-1.0071	-0.0866	2.1351
H	1.0283	0.7067	2.4630
C	-1.3136	-0.4325	0.9892
C	-2.6464	-0.1029	0.4522
C	-3.3060	1.0484	0.8913
C	-3.2515	-0.9260	-0.4997
C	-4.5553	1.3728	0.3645
H	-2.8523	1.6865	1.6426
C	-4.4855	-0.5916	-1.0387
H	-2.7541	-1.8354	-0.8512
C	-5.1400	0.5536	-0.6009
H	-5.0727	2.2665	0.7020
H	-4.9393	-1.2324	-1.7891
H	-6.1128	0.8042	-1.0162
C	-0.3313	-1.1489	0.1341
C	-0.2183	-0.8355	-1.2288
C	0.5543	-2.0501	0.7227
C	0.7754	-1.4326	-2.0031
H	-0.8513	-0.0812	-1.6889
C	1.5528	-2.6406	-0.0565
H	0.5121	-2.2442	1.7900
C	1.6641	-2.3248	-1.4115
H	0.8881	-1.1501	-3.0462
H	2.2801	-3.2968	0.4106
H	2.4734	-2.7491	-2.0024

Conformer 2

C	3.1806	0.8314	0.0595
C	1.9967	1.3104	0.6192
C	1.1069	2.1345	-0.0806
C	1.4315	2.4622	-1.4001
C	2.6015	1.9878	-1.9904
C	3.4873	1.1763	-1.2709
C	3.8228	0.0402	1.0521
C	3.0174	0.0313	2.1683
H	0.1888	2.4882	0.3765
H	0.7558	3.0906	-1.9750
H	2.8305	2.2475	-3.0206
H	4.3947	0.8068	-1.7380
H	4.7629	-0.4844	0.9491
H	3.1206	-0.4651	3.1231
N	1.9054	0.7836	1.8891
O	-1.0198	-0.1851	2.0618
H	1.0951	0.8817	2.4899
C	-1.3551	-0.5339	0.9306
C	-2.7104	-0.1911	0.4227
C	-3.3788	-1.0091	-0.4965
C	-3.3301	0.9704	0.8982

C	-4.6399	-0.6413	-0.9691
H	-2.9366	-1.9373	-0.8497
C	-4.5917	1.3327	0.4244
H	-2.8288	1.5976	1.6312
C	-5.2421	0.5311	-0.5138
H	-5.1549	-1.2716	-1.6908
H	-5.0683	2.2395	0.7886
H	-6.2245	0.8160	-0.8838
C	-0.3811	-1.2597	0.0768
C	0.4763	-2.1910	0.6706
C	-0.2459	-0.9526	-1.2822
C	1.4592	-2.8192	-0.0947
H	0.4045	-2.4011	1.7355
C	0.7377	-1.5859	-2.0447
H	-0.8593	-0.1881	-1.7500
C	1.5891	-2.5170	-1.4506
H	2.1449	-3.5208	0.3732
H	0.8644	-1.3245	-3.0922
H	2.3745	-2.9849	-2.0381

Conformer 3

C	1.3078	2.6835	1.2344
C	1.5073	1.9052	0.0946
C	2.2494	0.7181	0.1080
C	2.7866	0.3057	1.3308
C	2.5939	1.0565	2.4890
C	1.8624	2.2502	2.4541
C	0.5153	3.7993	0.8461
C	0.2285	3.6652	-0.4935
H	2.3818	0.1263	-0.7916
H	3.3549	-0.6200	1.3784
H	3.0117	0.7126	3.4316
H	1.7107	2.8269	3.3609
H	0.1702	4.5988	1.4874
H	-0.3651	4.2712	-1.1637
N	0.8158	2.5072	-0.9342
O	-0.6528	0.1154	-2.2375
H	0.6975	2.1009	-1.8550
C	-0.8783	-0.6759	-1.3255
C	-1.3275	-0.1613	-0.0054
C	-2.1724	0.9519	0.0322
C	-0.8377	-0.7063	1.1887
C	-2.5316	1.5119	1.2593
H	-2.5217	1.4071	-0.8916
C	-1.2015	-0.1444	2.4127
H	-0.1289	-1.5298	1.1805
C	-2.0459	0.9642	2.4470
H	-3.1623	2.3965	1.2873
H	-0.7935	-0.5470	3.3360

H	-2.3007	1.4212	3.3995
C	-0.6599	-2.1317	-1.5421
C	0.3398	-2.5348	-2.4347
C	-1.4374	-3.0945	-0.8866
C	0.5882	-3.8940	-2.6374
H	0.9305	-1.7937	-2.9678
C	-1.1823	-4.4524	-1.0890
H	-2.2467	-2.8056	-0.2203
C	-0.1676	-4.8502	-1.9585
H	1.3703	-4.2064	-3.3250
H	-1.7789	-5.1994	-0.5710
H	0.0295	-5.9076	-2.1136

Conformer 4

C	3.0474	0.7689	-0.0014
C	1.8942	1.2369	0.6271
C	0.9514	2.0450	-0.0220
C	1.1893	2.3694	-1.3606
C	2.3323	1.9148	-2.0157
C	3.2717	1.1190	-1.3471
C	3.7395	-0.0414	0.9420
C	2.9906	-0.0759	2.0966
H	0.0436	2.3641	0.4795
H	0.4600	2.9672	-1.9019
H	2.4917	2.1668	-3.0607
H	4.1474	0.7488	-1.8705
H	4.6607	-0.5819	0.7730
H	3.1284	-0.6129	3.0250
N	1.8703	0.6886	1.8923
O	-0.9960	-0.1709	2.1420
H	1.0732	0.7418	2.5171
C	-1.2962	-0.4935	0.9948
C	-2.6255	-0.1198	0.4396
C	-3.2433	1.0536	0.8795
C	-3.2505	-0.9262	-0.5167
C	-4.4745	1.4337	0.3296
H	-2.7734	1.6804	1.6293
C	-4.4702	-0.5406	-1.0698
H	-2.7961	-1.8554	-0.8492
C	-5.0785	0.6381	-0.6427
H	-4.9559	2.3499	0.6623
H	-4.9386	-1.1591	-1.8278
H	-6.0313	0.9371	-1.0711
C	-0.3175	-1.2237	0.1425
C	0.5563	-2.1349	0.7390
C	-0.1851	-0.9208	-1.2216
C	1.5563	-2.7386	-0.0259
H	0.4997	-2.3304	1.8071
C	0.8084	-1.5329	-1.9832

H	-0.8018	-0.1572	-1.6897
C	1.6816	-2.4366	-1.3829
H	2.2714	-3.4046	0.4494
H	0.9349	-1.2594	-3.0270
H	2.4887	-2.8738	-1.9639

Conformer 5

C	3.2414	0.7937	0.0867
C	2.0484	1.2969	0.6044
C	1.1914	2.1230	-0.1327
C	1.5588	2.4267	-1.4468
C	2.7387	1.9279	-1.9956
C	3.5919	1.1153	-1.2387
C	3.8424	0.0071	1.1084
C	3.0036	0.0239	2.1997
H	0.2648	2.4949	0.2917
H	0.9083	3.0545	-2.0507
H	3.0008	2.1684	-3.0226
H	4.5070	0.7264	-1.6739
H	4.7772	-0.5323	1.0403
H	3.0704	-0.4622	3.1631
N	1.9118	0.7888	1.8781
O	-1.0279	-0.1186	2.0354
H	1.0846	0.9049	2.4522
C	-1.3784	-0.4923	0.9157
C	-2.7511	-0.1950	0.4305
C	-3.4036	0.9440	0.9167
C	-3.4044	-1.0317	-0.4832
C	-4.6837	1.2645	0.4613
H	-2.9140	1.5868	1.6451
C	-4.6833	-0.7050	-0.9387
H	-2.9365	-1.9428	-0.8460
C	-5.3188	0.4443	-0.4710
H	-5.1852	2.1540	0.8340
H	-5.1850	-1.3497	-1.6568
H	-6.3144	0.6975	-0.8278
C	-0.4029	-1.2061	0.0541
C	-0.2786	-0.8909	-1.3049
C	0.4655	-2.1331	0.6392
C	0.7060	-1.5097	-2.0752
H	-0.9036	-0.1288	-1.7625
C	1.4492	-2.7484	-0.1359
H	0.4011	-2.3495	1.7027
C	1.5690	-2.4358	-1.4905
H	0.8250	-1.2403	-3.1213
H	2.1444	-3.4452	0.3245
H	2.3563	-2.8923	-2.0845

Conformer 6

C	1.4045	3.0402	-0.2571
C	1.8244	1.7223	-0.0800
C	1.8499	1.0971	1.1725
C	1.4126	1.8387	2.2738
C	0.9756	3.1538	2.1258
C	0.9721	3.7688	0.8677
C	1.5137	3.3315	-1.6453
C	1.9626	2.1949	-2.2790
H	2.1729	0.0672	1.2813
H	1.4051	1.3773	3.2582
H	0.6301	3.7092	2.9938
H	0.6255	4.7915	0.7588
H	1.2645	4.2661	-2.1290
H	2.1497	1.9820	-3.3223
N	2.1286	1.2216	-1.3274
O	0.8648	-1.5867	-1.4023
H	2.3597	0.2518	-1.5115
C	-0.1562	-1.5749	-0.7162
C	-0.9905	-0.3447	-0.6822
C	-1.4915	0.1502	0.5290
C	-1.1884	0.3726	-1.8655
C	-2.1919	1.3564	0.5511
H	-1.2951	-0.3614	1.4676
C	-1.8890	1.5790	-1.8377
H	-0.7646	0.0151	-2.8007
C	-2.3890	2.0699	-0.6311
H	-2.5495	1.7594	1.4951
H	-2.0136	2.1545	-2.7513
H	-2.9050	3.0261	-0.6066
C	-0.5269	-2.7638	0.0957
C	-1.8620	-3.0569	0.4039
C	0.4906	-3.6119	0.5464
C	-2.1704	-4.1671	1.1930
H	-2.6748	-2.4373	0.0338
C	0.1774	-4.7234	1.3326
H	1.5282	-3.4069	0.2926
C	-1.1508	-4.9951	1.6607
H	-3.2062	-4.3876	1.4388
H	0.9705	-5.3768	1.6878
H	-1.3924	-5.8586	2.2763

Conformer 7

C	1.2178	2.6227	1.2841
C	1.6261	1.7416	0.2833
C	2.2203	0.5043	0.5593
C	2.3837	0.1509	1.9018
C	1.9741	1.0056	2.9236
C	1.3953	2.2464	2.6294
C	0.6668	3.7640	0.6380

C	0.7251	3.5471	-0.7199
H	2.5235	-0.1656	-0.2383
H	2.8303	-0.8088	2.1499
H	2.1023	0.7077	3.9610
H	1.0738	2.9050	3.4299
H	0.2499	4.6382	1.1192
H	0.3904	4.1449	-1.5563
N	1.2867	2.3131	-0.9237
O	-0.0941	-0.0701	-2.3566
H	1.3735	1.8471	-1.8190
C	-0.6016	-0.7234	-1.4468
C	-1.3498	-0.0181	-0.3714
C	-2.0698	1.1352	-0.6969
C	-1.2620	-0.4289	0.9651
C	-2.7068	1.8671	0.3070
H	-2.1077	1.4854	-1.7257
C	-1.9023	0.3039	1.9655
H	-0.6535	-1.2831	1.2519
C	-2.6230	1.4508	1.6356
H	-3.2425	2.7787	0.0561
H	-1.8067	0.0008	3.0046
H	-3.0958	2.0370	2.4192
C	-0.4244	-2.1983	-1.4159
C	-1.4130	-3.0481	-0.9047
C	0.7591	-2.7420	-1.9317
C	-1.1975	-4.4268	-0.8666
H	-2.3565	-2.6527	-0.5378
C	0.9692	-4.1217	-1.8940
H	1.5213	-2.0920	-2.3561
C	-0.0055	-4.9614	-1.3552
H	-1.9615	-5.0849	-0.4598
H	1.8923	-4.5414	-2.2867
H	0.1603	-6.0357	-1.3241

Conformer 8

C	3.2994	0.7069	0.1289
C	2.0937	1.2419	0.5813
C	1.2803	2.0572	-0.2154
C	1.7040	2.3132	-1.5228
C	2.9012	1.7872	-2.0047
C	3.7123	0.9896	-1.1876
C	3.8340	-0.0770	1.1894
C	2.9411	-0.0324	2.2363
H	0.3345	2.4402	0.1533
H	1.0801	2.9174	-2.1766
H	3.2057	1.9877	-3.0285
H	4.6365	0.5721	-1.5741
H	4.7534	-0.6459	1.1695
H	2.9413	-0.5213	3.2006

N	1.8875	0.7594	1.8562
O	-1.0469	0.0007	2.0321
H	1.0279	0.8853	2.3798
C	-1.4083	-0.4041	0.9307
C	-2.8018	-0.1867	0.4644
C	-3.4004	-1.0534	-0.4643
C	-3.5267	0.9027	0.9610
C	-4.6907	-0.8025	-0.9257
H	-2.8737	-1.9290	-0.8369
C	-4.8223	1.1449	0.5022
H	-3.0802	1.5675	1.6973
C	-5.3983	0.2998	-0.4483
H	-5.1416	-1.4651	-1.6593
H	-5.3828	1.9965	0.8807
H	-6.4049	0.4939	-0.8099
C	-0.4239	-1.0820	0.0487
C	-0.3290	-0.7430	-1.3077
C	0.4764	-1.9922	0.6082
C	0.6647	-1.3153	-2.1006
H	-0.9825	0.0084	-1.7438
C	1.4732	-2.5570	-0.1887
H	0.4365	-2.2225	1.6701
C	1.5679	-2.2169	-1.5393
H	0.7660	-1.0196	-3.1411
H	2.2072	-3.2242	0.2554
H	2.3737	-2.6231	-2.1457

Conformer 9

C	2.4442	1.6312	-0.4663
C	2.4031	0.9809	0.7672
C	3.3552	0.0290	1.1518
C	4.3697	-0.2769	0.2393
C	4.4301	0.3492	-1.0041
C	3.4773	1.3086	-1.3676
C	1.3277	2.5119	-0.5136
C	0.6278	2.3656	0.6620
H	3.2964	-0.4737	2.1112
H	5.1147	-1.0243	0.5013
H	5.2197	0.0865	-1.7031
H	3.5249	1.7851	-2.3415
H	1.0539	3.1614	-1.3337
H	-0.2877	2.8199	1.0150
N	1.2797	1.4308	1.4257
O	-1.0278	-0.5421	2.1497
H	0.9431	1.0615	2.3066
C	-1.3945	-0.6861	0.9829
C	-2.7849	-0.3310	0.5906
C	-3.4401	0.6921	1.2847
C	-3.4483	-1.0041	-0.4442

C	-4.7325	1.0699	0.9157
H	-2.9424	1.2025	2.1063
C	-4.7375	-0.6188	-0.8142
H	-2.9771	-1.8304	-0.9703
C	-5.3758	0.4206	-0.1385
H	-5.2368	1.8720	1.4490
H	-5.2441	-1.1312	-1.6284
H	-6.3795	0.7211	-0.4301
C	-0.4274	-1.1831	-0.0297
C	-0.3872	-0.6366	-1.3196
C	0.5079	-2.1510	0.3483
C	0.5693	-1.0766	-2.2334
H	-1.0606	0.1656	-1.6100
C	1.4644	-2.5870	-0.5695
H	0.5098	-2.5451	1.3614
C	1.4938	-2.0511	-1.8577
H	0.6203	-0.6320	-3.2234
H	2.2099	-3.3189	-0.2702
H	2.2617	-2.3673	-2.5590

Conformer 10

C	1.8281	2.7680	-0.7487
C	1.7090	2.2968	0.5590
C	1.2409	3.0943	1.6110
C	0.8957	4.4168	1.3158
C	1.0121	4.9171	0.0203
C	1.4738	4.1034	-1.0214
C	2.2750	1.6799	-1.5491
C	2.4079	0.5831	-0.7296
H	1.1246	2.6957	2.6136
H	0.5238	5.0608	2.1093
H	0.7350	5.9473	-0.1878
H	1.5407	4.4924	-2.0324
H	2.4347	1.6894	-2.6183
H	2.6670	-0.4449	-0.9409
N	2.0586	0.9625	0.5430
O	0.4180	-1.1840	1.7246
H	1.9329	0.3259	1.3220
C	-0.3768	-1.4843	0.8404
C	-0.8415	-0.4557	-0.1199
C	-0.8385	-0.7120	-1.4977
C	-1.2110	0.8021	0.3615
C	-1.2126	0.2901	-2.3916
H	-0.5159	-1.6760	-1.8869
C	-1.5631	1.8064	-0.5376
H	-1.1587	1.0182	1.4243
C	-1.5639	1.5489	-1.9083
H	-1.1961	0.0982	-3.4607
H	-1.7781	2.8043	-0.1719

H	-1.8046	2.3492	-2.6074
C	-0.8772	-2.8824	0.7002
C	-2.1156	-3.1463	0.0986
C	-0.0903	-3.9391	1.1715
C	-2.5485	-4.4554	-0.0664
H	-2.7545	-2.3391	-0.2476
C	-0.5345	-5.2512	1.0112
H	0.8656	-3.7434	1.6505
C	-1.7542	-5.5133	0.3872
H	-3.5004	-4.6545	-0.5478
H	0.0744	-6.0772	1.3688
H	-2.0982	-6.5354	0.2596

Structures optimized at M06-2X/6-311++G(d,p)

Acetone-indole

C	-2.0163000	-0.4781000	-0.1696000
C	-0.8199000	-0.1717000	0.4776000
C	-0.4458000	1.1394000	0.7961000
C	-1.3160000	2.1688000	0.4278000
C	-2.5191000	1.8931000	-0.2187000
C	-2.8842000	0.5744000	-0.5192000
C	-2.0557000	-1.8913000	-0.3275000
C	-0.8931000	-2.4061000	0.1999000
H	0.4891000	1.3513000	1.3045000
H	-1.0502000	3.1990000	0.6526000
H	-3.1846000	2.7084000	-0.4913000
H	-3.8252000	0.3683000	-1.0198000
H	-2.8466000	-2.4664000	-0.7899000
H	-0.5236000	-3.4205000	0.2659000
N	-0.1535000	-1.3602000	0.6872000
O	2.6604000	-0.6900000	0.6875000
H	0.7786000	-1.4286000	1.0862000
C	2.8546000	0.0915000	-0.2479000
C	3.7550000	1.2868000	-0.1049000
H	3.2138000	2.1891000	-0.4009000
H	4.0691000	1.3930000	0.9365000
H	4.6374000	1.1556000	-0.7349000
C	2.1632000	-0.0837000	-1.5726000
H	1.2920000	0.5742000	-1.6185000
H	2.8503000	0.1581000	-2.3864000
H	1.8466000	-1.1235000	-1.6869000

2-butanone-indole

C	2.3900000	-0.4700000	0.0510000
C	1.0850000	-0.2092000	0.4694000
C	0.5916000	1.0874000	0.6493000
C	1.4591000	2.1485000	0.3789000
C	2.7700000	1.9165000	-0.0381000

C	3.2502000	0.6134000	-0.2033000
C	2.5328000	-1.8799000	-0.0185000
C	1.3277000	-2.4428000	0.3378000
H	-0.4264000	1.2657000	0.9766000
H	1.1091000	3.1688000	0.5019000
H	3.4274000	2.7577000	-0.2350000
H	4.2726000	0.4415000	-0.5244000
H	3.4222000	-2.4248000	-0.3066000
H	1.0119000	-3.4738000	0.4057000
N	0.4569000	-1.4246000	0.6289000
O	-2.3752000	-0.9479000	0.3267000
H	-0.5227000	-1.5344000	0.8762000
C	-2.4992000	-0.1945000	-0.6368000
C	-3.4741000	0.9666000	-0.6636000
H	-2.9338000	1.8592000	-0.9972000
H	-4.2504000	0.7326000	-1.3992000
C	-1.6311000	-0.3495000	-1.8610000
H	-0.7628000	0.3037000	-1.7654000
H	-1.3105000	-1.3894000	-1.9533000
H	-2.1933000	-0.0726000	-2.7535000
C	-4.1054000	1.2367000	0.6878000
H	-3.3477000	1.5181000	1.4259000
H	-4.8300000	2.0536000	0.6123000
H	-4.6271000	0.3498000	1.0632000

2-pentanone-indole

C	2.1389000	-0.6725000	-0.3664000
C	1.2321000	-0.5227000	0.6827000
C	1.3008000	0.5369000	1.5953000
C	2.3165000	1.4806000	1.4168000
C	3.2317000	1.3609000	0.3728000
C	3.1580000	0.2869000	-0.5225000
C	1.7669000	-1.8470000	-1.0768000
C	0.6482000	-2.3687000	-0.4685000
H	0.5831000	0.6328000	2.4036000
H	2.3902000	2.3216000	2.1022000
H	4.0117000	2.1081000	0.2502000
H	3.8742000	0.2009000	-1.3338000
H	2.2581000	-2.2554000	-1.9497000
H	0.0410000	-3.2324000	-0.7021000
N	0.3246000	-1.5555000	0.5873000
O	-2.4455000	-0.8530000	1.1215000
H	-0.5032000	-1.6500000	1.1682000
C	-2.9479000	0.0634000	0.4650000
C	-2.0966000	1.1649000	-0.1285000
H	-2.6695000	2.0991000	-0.1360000
H	-1.2468000	1.3289000	0.5408000
C	-4.4298000	0.1363000	0.2126000
H	-4.9063000	-0.7931000	0.5343000
H	-4.8523000	0.9712000	0.7763000

H	-4.6177000	0.2723000	-0.8551000
C	-1.6366000	0.7932000	-1.5319000
H	-2.4941000	0.7851000	-2.2140000
H	-1.2256000	-0.2221000	-1.5436000
C	-0.5886000	1.7649000	-2.0506000
H	-0.9940000	2.7783000	-2.1158000
H	0.2872000	1.7888000	-1.3957000
H	-0.2555000	1.4649000	-3.0493000

Cyclohexanone-indole

C	2.2417000	-0.1205000	0.4330000
C	1.4539000	-0.3415000	-0.6962000
C	1.0625000	0.6873000	-1.5602000
C	1.4790000	1.9850000	-1.2494000
C	2.2572000	2.2385000	-0.1217000
C	2.6506000	1.1949000	0.7250000
C	2.4319000	-1.3814000	1.0632000
C	1.7499000	-2.3250000	0.3290000
H	0.4449000	0.4893000	-2.4307000
H	1.1831000	2.8089000	-1.8947000
H	2.5632000	3.2567000	0.1052000
H	3.2558000	1.4008000	1.6025000
H	2.9934000	-1.5719000	1.9680000
H	1.6209000	-3.3891000	0.4727000
N	1.1579000	-1.6866000	-0.7295000
O	-1.7574000	-1.6145000	-1.3159000
H	0.5307000	-2.1147000	-1.4015000
C	-2.1335000	-0.8201000	-0.4509000
C	-2.8854000	0.4438000	-0.8082000
C	-2.0091000	-1.1326000	1.0232000
C	-2.3725000	1.6282000	-0.0004000
H	-3.9492000	0.2715000	-0.6091000
H	-2.7656000	0.6433000	-1.8778000
C	-1.5039000	0.0806000	1.7912000
H	-2.9954000	-1.4398000	1.3874000
H	-1.3218000	-1.9741000	1.1572000
C	-2.3292000	1.3302000	1.4962000
H	-3.0072000	2.5024000	-0.1846000
H	-1.3659000	1.8934000	-0.3402000
H	-1.5206000	-0.1304000	2.8655000
H	-0.4606000	0.2771000	1.5244000
H	-1.8954000	2.1864000	2.0258000
H	-3.3487000	1.1978000	1.8769000

Acetophenone-indole

C	1.9980470	-0.0829540	0.1571130
C	1.0770290	-0.9910080	0.7320230
C	0.5583650	-2.0788290	0.0240540
C	-1.4334369	0.9557680	-2.2299120

C	1.8603250	-1.3298980	-1.8902480
C	2.3889540	-0.2698420	-1.1772910
C	2.3075380	0.8875460	1.1690210
C	1.5731640	0.5561950	2.2706250
H	-0.1285650	-2.7741040	0.4941570
H	0.5750280	-3.0631680	-1.8723060
H	2.1522030	-1.4821740	-2.9225710
H	3.0903110	0.4131400	-1.6432460
H	2.9813980	1.7249630	1.0842750
H	1.5084050	1.0450290	3.2301670
N	0.8435910	-0.5854530	2.0229790
O	-2.1468070	-0.8735750	1.6893020
H	0.0646300	-0.9087150	2.5781950
C	-2.2009140	-0.6782260	0.4919710
C	-2.9078890	-1.6533090	-0.4201010
H	-3.6354200	-1.1356510	-1.0487470
H	-2.1799810	-2.1336510	-1.0792540
H	-3.4030140	-2.4088700	0.1862640
C	-1.5573580	0.5273050	-0.1160610
C	-1.2578850	0.5878530	-1.4787220
C	-1.1968780	1.5857310	0.7193140
C	-0.5848830	1.6883420	-1.9942000
H	-1.5127680	-0.2364670	-2.1337730
C	-0.5401710	2.6915200	0.1996410
H	-1.4312490	1.5180020	1.7750870
C	-0.2271810	2.7398560	-1.1562590
H	-0.3296230	1.7199630	-3.0465330
H	-0.2575880	3.5092930	0.8517520
H	0.2993750	3.5969510	-1.5598390

Benzophenone-indole

C	-2.8183270	-0.9843980	-0.2497770
C	-1.6858050	-1.3483820	0.5168770
C	-0.5810020	-1.9967100	-0.0431670
C	-0.6148390	-2.2563000	-1.4011330
C	-1.7275930	-1.8940440	-2.1860910
C	-2.8265820	-1.2716620	-1.6232290
C	-3.7366900	-0.3429190	0.6488110
C	-3.1397020	-0.3268540	1.8763330
H	0.2706950	-2.2792930	0.5652490
H	0.2279040	-2.7519930	-1.8689470
H	-1.7210850	-2.1174880	-3.2464330
H	-3.6813080	-1.0029090	-2.2339610
H	-4.7102370	0.0552240	0.4113750
H	-3.4933480	0.0812490	2.8103390
N	-1.9141320	-0.9506640	1.8117680
O	0.8421050	0.0719090	2.1657260
H	-1.1791040	-0.8603570	2.4992650
C	1.1204070	0.4846370	1.0543990
C	2.4671390	0.1930940	0.4690640

C	3.1328230	-0.9608020	0.8889480
C	3.0918580	1.0639920	-0.4256420
C	4.3961180	-1.2566010	0.3973710
H	2.6453990	-1.6114210	1.6056350
C	4.3673000	0.7773300	-0.9000040
H	2.5897180	1.9734000	-0.7336830
C	5.0146530	-0.3861820	-0.4977140
H	4.9035650	-2.1596250	0.7147740
H	4.8551590	1.4622320	-1.5829950
H	6.0043910	-0.6129650	-0.8764320
C	0.1206090	1.2596190	0.2598640
C	0.0539850	1.1583730	-1.1325800
C	-0.8416070	2.0004300	0.9482110
C	-0.9703910	1.7911860	-1.8248280
H	0.7672350	0.5418220	-1.6660720
C	-1.8508570	2.6497140	0.2514400
H	-0.7894190	2.0480040	2.0298460
C	-1.9174300	2.5419670	-1.1349460
H	-1.0394160	1.6823750	-2.9003750
H	-2.5960810	3.2241550	0.7883770
H	-2.7164000	3.0340430	-1.6773830
